# Molecular mechanisms regulating differential expression of individual miRNAs within polycistronic clusters

**DOI:** 10.3389/fmolb.2026.1913118

**Published:** 2026-10-08

**Authors:** Diana Maltseva, Alexander Tonevitsky

**Affiliations:** 1 Faculty of Biology and Biotechnology, National Research University Higher School of Economics, Moscow, Russia; 2 Shemyakin-Ovchinnikov Institute of Bioorganic Chemistry, Russian Academy of Sciences, Moscow, Russia

**Keywords:** alternative promoter, alternative splicing, epitranscriptome, epitranscriptomic RNA modifications, miRNA biogenesis, miRNA polycistronic clusters, RNA-binding proteins

## Abstract

MicroRNA (miRNA) genes are frequently located in close proximity within the genome, forming so-called clusters. These clusters are transcribed as polycistronic units, suggesting coordinated transcriptional regulation of the miRNAs they contain. However, individual mature miRNAs from the same cluster often accumulate at varying levels and exhibit distinct tissue-specific expression patterns, indicating the presence of regulatory mechanisms that modulate the differential expression of individual cluster members. This review summarizes the molecular mechanisms underlying the differential regulation of individual miRNAs encoded within polycistronic transcripts. These mechanisms range from strict genomic and transcriptional controls to dynamic post-transcriptional events. At the genomic and transcriptional levels, the use of alternative promoters enables independent transcription of specific cluster members, while alternative splicing can either facilitate or disrupt the processing of precursor hairpins. At the post-transcriptional level, epitranscriptomic modifications (including A-to-I editing, m^6^A methylation, m^5^C, m^7^G, and others) selectively modulate Drosha- and Dicer-mediated cleavage, target binding, and miRNA stability. Furthermore, precursor processing is governed by the structural characteristics of neighboring hairpins through a process termed “cluster assistance” and by RNA-binding proteins (RBPs) that recognize specific hairpin motifs to regulate biogenesis in a context-dependent manner. Understanding these diverse regulatory layers provides critical insight into how cells fine-tune the coordinated versus differential expression of miRNA clusters to achieve distinct physiological and pathological outcomes.

## 1 Introduction

MicroRNAs (miRNAs) are small non-coding RNAs of approximately 22 nucleotides in length (Gebert and MacRae, 2018). By binding complementary sequences through their seed region (nucleotides 2–7/8 at the 5′end of the miRNA) or via full-sequence complementarity to target mRNAs, miRNAs induce mRNA deadenylation, translational repression, and/or mRNA degradation (Gebert and MacRae, 2018). MiRNAs are important regulators of gene expression, and their dynamic regulation is essential for normal physiological processes, including tissue differentiation (Esau et al., 2004; Schickel et al., 2008; Ivey and Srivastava, 2010), hematopoiesis (Chen et al., 2004), organismal development (Kloosterman and Plasterk, 2006; Mogilyansky and Rigoutsos, 2013), and apoptosis (Skommer et al., 2014; Su et al., 2015). Conversely, dysregulation of miRNAs is associated with various diseases and the aging process (Tang et al., 2012; Baranova et al., 2019; Di Martino et al., 2022; Maltseva et al., 2025). Approximately 60% of protein-coding genes are predicted to be miRNA targets (Friedman et al., 2009), highlighting the importance of precise miRNA regulation in both physiological and pathophysiological contexts.

Canonical miRNA biogenesis begins with the transcription of primary miRNA (pri-miRNA) by RNA polymerase II (RNA Pol II) (Lee et al., 2004) (Figure 1). Pri-miRNAs undergo sequential processing by two type III ribonucleases (RNase III), Drosha (within the Microprocessor complex) and Dicer, generating an RNA duplex of approximately 22 nucleotides (Shang and Lai, 2023). This duplex is subsequently loaded onto an Argonaute (Ago) family protein, which retains one strand as the guide and discards the other passenger strand, thereby generating the effector complex known as the RNA-induced silencing complex (RISC) (Grishok et al., 2001; Hammond et al., 2001). Non-canonical biogenesis pathways may bypass either Drosha- or Dicer-mediated steps, utilizing alternative mechanisms for miRNA generation (Bartel, 2018; Shang and Lai, 2023). The detailed steps of canonical and non-canonical miRNA biogenesis have been extensively reviewed elsewhere (Treiber et al., 2018; Vilimova and Pfeffer, 2023; Kim et al., 2025). Therefore, we focus here only on general features of miRNA biogenesis (Figure 1) and on selected mechanisms particularly relevant to this review. One such feature is alternative cleavage of miRNA precursors by Drosha or Dicer. Cleavage can occur at alternative positions, shifting the processing site by one or more nucleotides toward either the 5′or 3′end (Gebert and MacRae, 2018). This alternative processing generates miRNA isoforms (isomiRs), more precisely template-dependent isomiRs, that differ from their canonical counterparts at their 5′and/or 3′termini. Notably, even a single-nucleotide shift at the 5′end alters the seed region, which radically reprograms the spectrum of mRNA targets the miRNA can interact with. Alternative 5′end processing is considerably more frequent during Dicer-mediated cleavage (42%) compared to Drosha-mediated cleavage (14%) (Zhiyanov et al., 2021). Recent studies have investigated the mechanisms governing the accuracy of pre-miRNA cleavage by Dicer. For instance, a GYM motif (comprising a paired guanine [G], a paired pyrimidine [Y], and a mismatched cytosine or adenine [M]) positioned within the cleavage site has been shown to promote more precise and homogeneous processing (Lee et al., 2023). However, the GYM motif alone does not always guarantee cleavage homogeneity (Maltseva et al., 2024), indicating that other structural or sequence features of the pre-miRNA are involved. Specifically, evidence highlights the important role of the nucleotides flanking the GYM motif (Zhiyanov et al., 2021; Le et al., 2024). Thus, the exact mechanisms underlying alternative Dicer-mediated processing remain incompletely understood.

**FIGURE 1:**
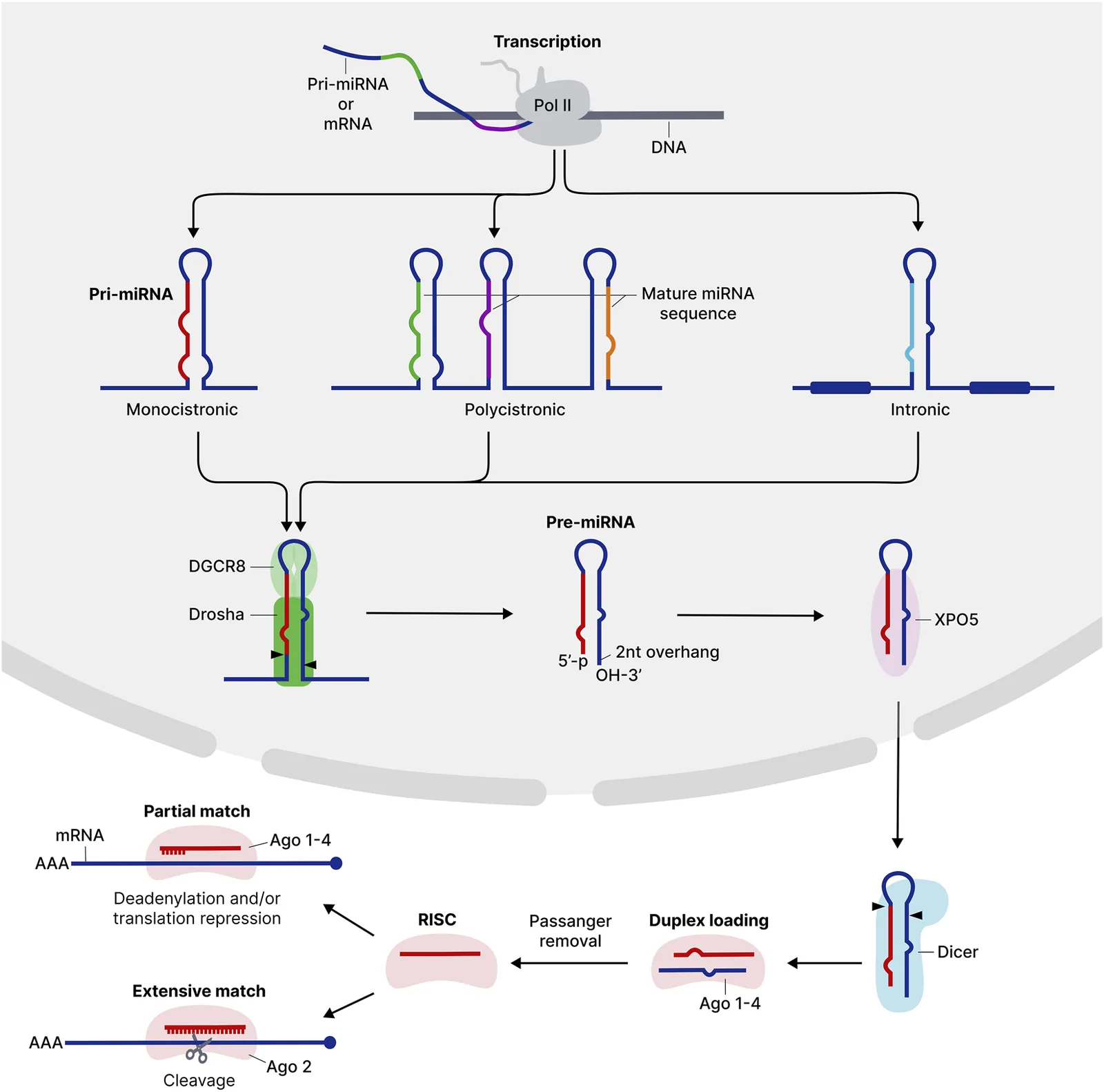
Canonical miRNA biogenesis. In animals, miRNAs are encoded either as independent genes (monocistronic), as part of gene clusters (polycistronic), or within host genes, predominantly in introns. In the cell nucleus, RNA polymerase II (Pol II) transcribes primary miRNA transcripts (pri-miRNAs), which contain hairpin structures (one per monocistronic gene or multiple in polycistronic clusters) flanked by 5′and 3′sequences. The Microprocessor complex, consisted of a DGCR8 dimer and the endonuclease Drosha, cleaves pri-miRNA within the stem region of each hairpin (black arrows), generating precursor miRNAs (pre-miRNAs) characterized by 3′-hydroxyl group (OH-3′), a 2-nucleotide 3′overhang (2 nt overhang), and a 5′-phosphate group (5′-p). Exportin 5 (XPO5) binds pre-miRNAs and facilitates their transport from the nucleus to the cytoplasm. In the cytoplasm, Dicer cleaves pre-miRNAs near the terminal loop (black arrows), producing a miRNA duplex. The duplex is then loaded onto Argonaute (Ago) proteins (Ago1–4 in humans), which select only one strand to form the RNA-induced silencing complex (RISC). Upon RISC assembly, the mature miRNA guides the complex to its target mRNA through complementary base pairing, predominantly mediated by the miRNA seed region (nucleotides 2–7/8). In cases of partial complementarity, with some mismatches at the center of the miRNA, the RISC mediates translational repression and/or deadenylation of the target transcript. Conversely, when base pairing is extensive, catalytically active Ago2 executes endonucleolytic cleavage of the target mRNA between nucleotides 10 and 11, thereby triggering its degradation. During biogenesis, Dicer and Drosha can cleave miRNA precursors at alternative positions, shifting the cleavage site by one or more nucleotides toward the 5′or 3′end. This leads to the formation of template-dependent miRNA isoforms (not indicated in the figure).

According to various estimates, 18.7%–42% of human miRNA loci are organized into genomic clusters—groups of miRNA genes located in close proximity on the same DNA strand (Altuvia et al., 2005; Wang et al., 2016; Kabekkodu et al., 2018; Ware et al., 2022). This wide divergence in estimates stems primarily from two factors: first, differences in the number of annotated miRNA loci at the time of each study, and second, the lack of a standardized operational definition, with genomic distance cutoffs ranging from conservative thresholds of <1–3 kb (Altuvia et al., 2005) to intermediate windows of 10 kb (Wang et al., 2016) or broad spans up to 50 kb (Baskerville and Bartel, 2005; Liang et al., 2007). Biologically, setting an inter-hairpin distance cutoff serves as a practical proxy to ensure (or at least maximize the probability of) cotranscription within a single polycistronic unit. Genome-wide analyses reveal that inter-miRNA distances follow a bimodal distribution with a local minimum near 10 kb (Shalgi et al., 2007). This 10-kb boundary is further corroborated by nascent RNA sequencing and expressed sequence tag (EST) datasets confirming shared transcription units (Saini et al., 2007). Conversely, expanding the threshold up to 50 kb risks overestimating cluster sizes by conflating independent transcription units, whereas stringent limits of ≤3 kb systematically exclude distal yet genuine cotranscribed members (Altuvia et al., 2005). Clusters are co-transcribed from a single promoter as a polycistronic pri-miRNA molecule, which possesses a 5′cap and a 3′poly(A) tail, characteristic of RNA Pol II transcripts. miRNA clusters comprise from two to several dozen genes, most commonly two to six. The largest human cluster, C19MC, contains 46 miRNAs. According to (Ware et al., 2022), humans have 159 clusters, which predominantly consist of evolutionarily conserved miRNAs (Wang et al., 2016).

Clusters are named using different conventions. One approach lists the miRNAs in transcriptional order, separating by a tilde (∼), e.g., the miR-30a∼miR-30c-2 cluster. Another method indicates the first and last miRNA within the cluster, e.g., the miR-17–92 cluster (also denoted as miR-17/92 or miR-17–92), which contains six miRNAs (Desvignes et al., 2015). A third nomenclature designates clusters as miRc-X (*MIRCX* gene), where “c” denotes cluster and X corresponds to the first miRNA in the cluster (Desvignes et al., 2015); however, this system is rarely used.

According to recent models of miRNA evolution, genomic clustering originally arose as an adaptive mechanism to drive the synergistic repression of shared biological pathways. Beyond the transcriptional economy of cotranscribing multiple regulators from a single locus, clustered miRNAs frequently converge functionally to target common signaling cascades or cellular processes (Kim et al., 2009; Wang et al., 2016; Li et al., 2021), indicating the functional significance of miRNA clustering. The synergistic action of cluster members allows for more potent and robust suppression of a biological process than the independent action of individual miRNAs.

Moreover, from an evolutionary standpoint, operonic organisation promotes the emergence of novel miRNAs. Clustering has permitted cells to salvage and express evolutionarily young and weak (suboptimal, i.e., characterised by suboptimal processing structure and/or sequence) pri-miRNA hairpins by virtue of synergy with robust hairpins (Shang et al., 2020). The elevated local concentration of the Microprocessor complex resulting from clustering facilitates the recognition of suboptimal substrates.

Despite being transcribed together, mature miRNAs from the same cluster can accumulate at different levels and exhibit distinct tissue-specific and stimulus-dependent expression patterns (Contrant et al., 2014; Baldrich et al., 2016; Truscott et al., 2016; Wang et al., 2016). The emergence of complex tissue-specific programs required an evolutionary transition from rigid co-activation to dynamic decoupling of individual cluster members. Fine-tuning intra-cluster expression ratios is critical throughout development; for instance, the *Drosophila* let-7-C cluster undergoes stage-specific differential processing during metamorphosis (Chawla and Sokol, 2014), while dysregulation of miR-998 within the miR-11∼miR-998 cluster triggers severe developmental defects (Truscott et al., 2016).

Furthermore, polycistronic miRNAs can exert directly opposing actions within the same tissue. In skeletal muscle, cotranscribed miR-1 and miR-133a act antagonistically: miR-1 drives differentiation and limits proliferation by suppressing HDAC4, whereas miR-133 promotes myoblast proliferation by repressing SRF (Chen et al., 2005). In cardiomyocytes, however, miR-133a acts context-dependently to inhibit proliferation, preserve differentiation, and repress cardiac hypertrophy (Carè et al., 2007). This functional plasticity reflects cell-type-specific mRNA target landscapes that tailor the physiological output of individual cluster members.

This dual nature is exemplified by the well-characterized miR-17–92 cluster. Depending on cellular context, mutational landscape, and the prevailing target transcriptome, this polycistron can function as either an oncogene or a tumor suppressor (Zhao et al., 2022). This functional duality arises because individual cluster members frequently target oncogenes in certain cell types while suppressing tumor suppressors in others. For example, miR-18a acts as a tumor suppressor by attenuating proliferation in colorectal cancer (via Cdc42) and bladder cancer (via Dicer1), yet it exerts clear oncogenic effects in pancreatic ductal adenocarcinoma and lymphomas (Zhao et al., 2022). Conversely, miR-92a is markedly elevated in colon cancer relative to other cluster members, where it exhibits potent oncogenic activity by downregulating the pro-apoptotic tumor suppressor BCL2L11 (BIM) (Tsuchida et al., 2011). Functional antagonism can also occur between cotranscribed members within the same cell type. In p53-intact pre-malignant B cells and embryonic fibroblasts from Eμ-myc mice, miR-19a/b-1 and miR-92a exert opposing effects on cell survival: miR-19a/b-1 suppresses apoptosis by targeting the tumor suppressor Pten, whereas miR-92a counteracts this survival signal by triggering apoptosis (Zeitels and Mendell, 2013). Paradoxically, miR-92a overexpression also enhances cell proliferation. This dual phenotype occurs because miR-92a directly represses Fbw7, an ubiquitin ligase responsible for targeting MYC for proteasomal degradation. The resulting stabilization and accumulation of MYC drives rapid proliferation while simultaneously activating the p53 pathway to promote apoptosis (Tsuchida et al., 2011). Notably, the expression of miR-19a/b-1 substantially exceeds that of miR-92a in both premalignant and transformed Eμ-myc B cells and human Burkitt lymphoma cell lines. Thus, the differential stoichiometric expression of miR-19a/b-1 relative to miR-92a ultimately dictates the net oncogenic output of the miR-17–92 cluster in p53-competent B-cell lymphomas (Tsuchida et al., 2011).

The miR-106b–25 cluster is a paralog of the miR-17–92 cluster and encodes three mature miRNAs: miR-106b, miR-93, and miR-25 (Zhao et al., 2022). In prostate cancer, elevated expression of miR-93-5p correlates strongly with disease aggressiveness (Zhiyanov et al., 2023). Mechanistically, miR-93 exerts oncogenic activity by repressing the tumor suppressors FRMD6 (Sun et al., 2023) and DAB2 (Yang et al., 2019), as well as by modulating TGFBR2, ITGB8, and LATS2 (Liu et al., 2018). In pancreatic ductal adenocarcinoma, which is characterized by pronounced immunosuppression, miR-106b and miR-93 (which share an identical seed sequence) directly target and downregulate CD274 (PD-L1) in both tumor cells and tumor microenvironment-associated myeloid-derived suppressor cells (Cioffi et al., 2017). Consequently, elevated levels of miR-106b and miR-93 can counteract tumor-mediated immune evasion by restoring antitumor immunity. Furthermore, in the bone marrow stroma, miR-106b and miR-93 suppress expression of the chemokine CXCL12 via direct 3′UTR binding, thereby disrupting pre-metastatic niche formation and curtailing distant metastasis (Cioffi et al., 2017). Restoring or pharmacologically enhancing miR-106b and miR-93 expression may therefore offer an effective therapeutic strategy in pancreatic cancer.

Together, these examples highlight the functional imperative of elucidating how individual miRNAs within polycistronic clusters are differentially regulated. Perturbations in the stoichiometric expression of clustered miRNAs frequently correlate with diverse human pathologies. In this review, we synthesize and conceptualize the molecular mechanisms underlying intra-cluster “decoupling” that enable cells to uncouple individual hairpin maturation and bypass the evolutionarily hardwired co-expression of polycistronic transcripts. These mechanisms mainly involve post-transcriptional regulation of miRNA processing and stability. Other regulatory possibilities include transcription from an alternative promoter and splicing of the host gene transcript.

## 2 Alternative promoters within miRNA clusters

Contrary to expectations, clustered miRNAs are not always co-transcribed. In several clusters, individual miRNAs can be expressed independently, indicating that a cluster may be regulated by more than one promoter. A notable example is the conserved miRNA cluster miR-100∼let-7a-2∼miR-125b-1 (Chang et al., 2015). In addition to the primary transcript, MIR100HG, generated from the distal promoter, a transcript encoding only miR-125b-1 is produced via proximal promoter (Figure 2A). The usage frequency of proximal promoter varies among cell lines, suggesting tissue-specific and signal-dependent regulation of alternative promoter activity.

**FIGURE 2:**
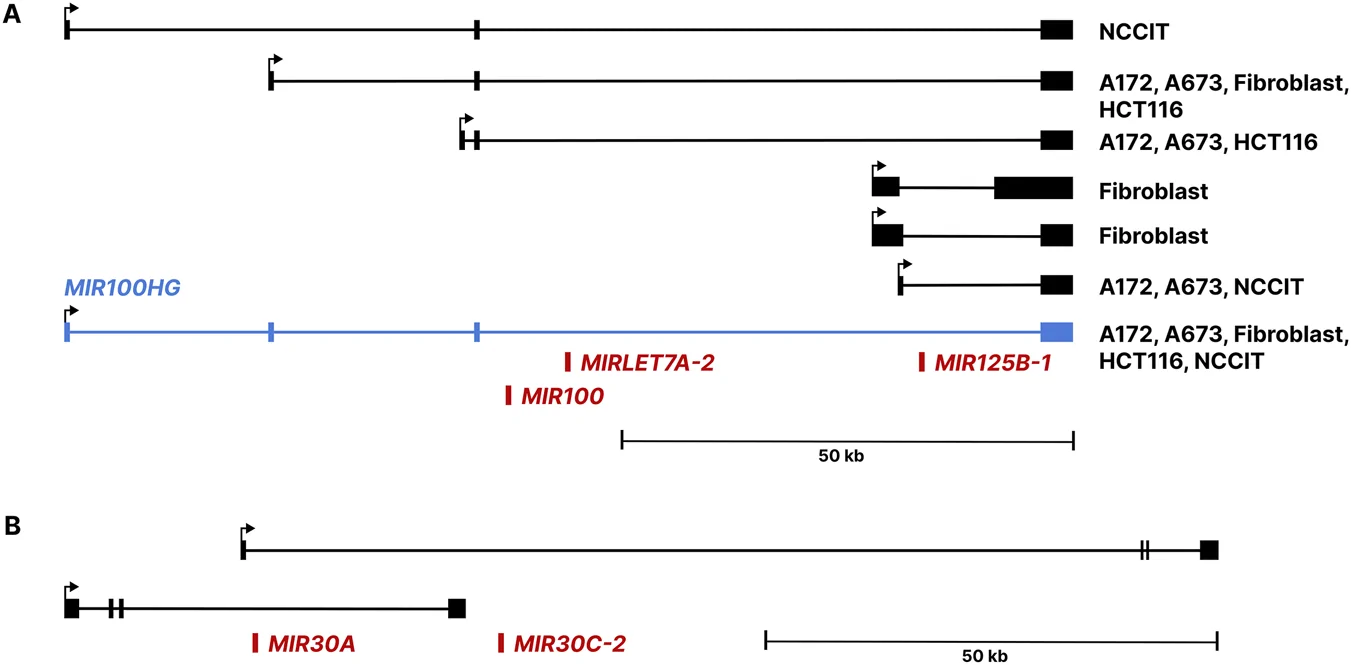
Alternative promoters enable the independent expression of clustered miRNAs. **(A)** Schematic representation of the miR-100∼let-7a-2∼miR-125b-1 cluster. The presence of an alternative promoter permits the independent expression of miR-125b-1, with the frequency of alternative promoter usage varying across cell lines. **(B)** Schematic representation of the miR-30a∼miR-30c-2 cluster. Transcription from the upstream promoter produces a transcript encoding exclusively miR-30a.

Another example is the miR-30a∼miR-30c-2 cluster (Chang et al., 2015). Transcription from the upstream promoter produces a transcript encoding miR-30a but not miR-30c-2. This finding suggests that under certain conditions, the expression of miR-30a can be uncoupled from that of miR-30c-2 (Figure 2B).

To date, there is no precise estimate of how many human miRNA clusters harbor internal alternative promoters enabling independent expression of individual cluster members; only isolated examples have been described, and no systematic registry exists. In the study by (Chang et al., 2015), 21 intergenic clusters were found to contain alternative promoters. In addition to those already mentioned, alternative promoters have been identified in well-known intergenic oncogenic cluster as miR-221∼miR-222 (Di Martino et al., 2022). According to Bouvy-Liivrand et al., approximately 20% of miRNA clusters display evidence of multiple TSS (Bouvy-Liivrand et al., 2017).

For the miR-106b∼miR-93∼miR-25 cluster, encoded within intron 13 of the MCM7 gene, an additional intronic promoter produces a transcript containing only pri-miR-106b and pri-miR-93 but not pri-miR-25 (Ramalingam et al., 2014). It is estimated that about half of human miRNAs (49%) are located within introns of host genes, predominantly protein-coding genes (Liu et al., 2021). Approximately 35% of these intronic miRNAs contain their own intragenic transcription start sites (TSSs) (Ramalingam et al., 2014). The extent to which these intragenic promoters correspond to clustered miRNAs, and how many clusters contain additional alternative promoters, remains an open question.

As observed for the miR-100∼let-7a-2∼miR-125b-1 cluster, the selection of alternative transcription start sites (TSSs) is highly cell-type-specific. By integrating CAGE-seq, GRO-seq, and histone modification profiles (H3K4me3/H3K4me1) across 27 cell types, Bouvy-Liivrand et al. demonstrated that lineage-specific and stimulus-responsive TSS usage is a widespread feature of polycistronic miRNA loci (Bouvy-Liivrand et al., 2017). Crucially, super-enhancers (SEs) within or proximal to these loci are major drivers of alternative TSS selection. For example, at the miR-221∼miR-222 locus, adjacent SE constituents selectively engage distinct TSSs across different cellular lineages; among these, enhancer element SE4 contains an NF-κB binding site that drives rapid, selective TSS activation upon TNFα stimulation (Bouvy-Liivrand et al., 2017). Chromatin conformation analyses further indicate that physical interactions between SEs and specific TSSs within the miR-221∼miR-222 locus are constrained within topologically associating domains (TADs). TADs are insulated genomic neighborhoods demarcated by CTCF-binding sites and insulator elements (Bouvy-Liivrand et al., 2017). Thus, 3D chromatin architecture provides a fundamental structural scaffold that defines the regulatory boundaries of miRNA clusters and governs their cell-type-specific and inducible transcriptional output.

For intronic miRNA clusters, nuclear matrix attachment regions (MARs) serve as critical cis-regulatory elements. Flanking MAR sequences within the miR-17–92 locus act as structural chromatin domain boundaries: in IMR-32 neuroblastoma cells, where the cluster is robustly transcribed, histone H3 across both the cluster and its adjacent MARs is hyperacetylated at lysines 9 and 14 (H3K9/14ac) (Chavali et al., 2011). In contrast, in primary mouse embryonic fibroblasts where the locus is silenced, these identical MARs are enriched for the repressive heterochromatin mark H3K9me3 (Chavali et al., 2011). Recruitment of the architectural high-mobility group protein HMGA1 (HMG I/Y) to these MAR elements promotes localized histone hyperacetylation, facilitating chromatin decompaction into an open conformation permissive for transcription. This demonstrates that for intronic clusters, transcriptional competence in a given cellular context is governed by the 3D chromatin architecture of the locus rather than basal promoter accessibility alone.

## 3 Regulation of miRNA biogenesis through alternative splicing

As noted above, most human miRNAs are encoded within introns of host genes (Liu et al., 2021), and their expression generally correlates with that of the host gene. Approximately 4% of miRNAs reside within exons, and about 24% are located in intergenic regions. Additionally, a small subset of miRNAs (2.4%) has pre-miRNA sequences spanning intron-exon junctions (Liu et al., 2021). In such cases, splicing of the host gene primary transcripts can interfere with precursor hairpin processing by the Microprocessor complex (Drosha/DGCR8) (Figure 3A). This has been demonstrated for miR-205 (Chang et al., 2015), whose pre-miR-205 hairpin overlaps a splice site immediately upstream of the last exon of the annotated *MIR205HG* gene. Utilization of this splice site disrupts the precursor hairpin, making it mutually exclusive with mature miRNA production. Conversely, alternatively spliced isoforms that place the pre-miR-205 hairpin entirely within an intron favor miRNA processing (Chang et al., 2015).

**FIGURE 3:**
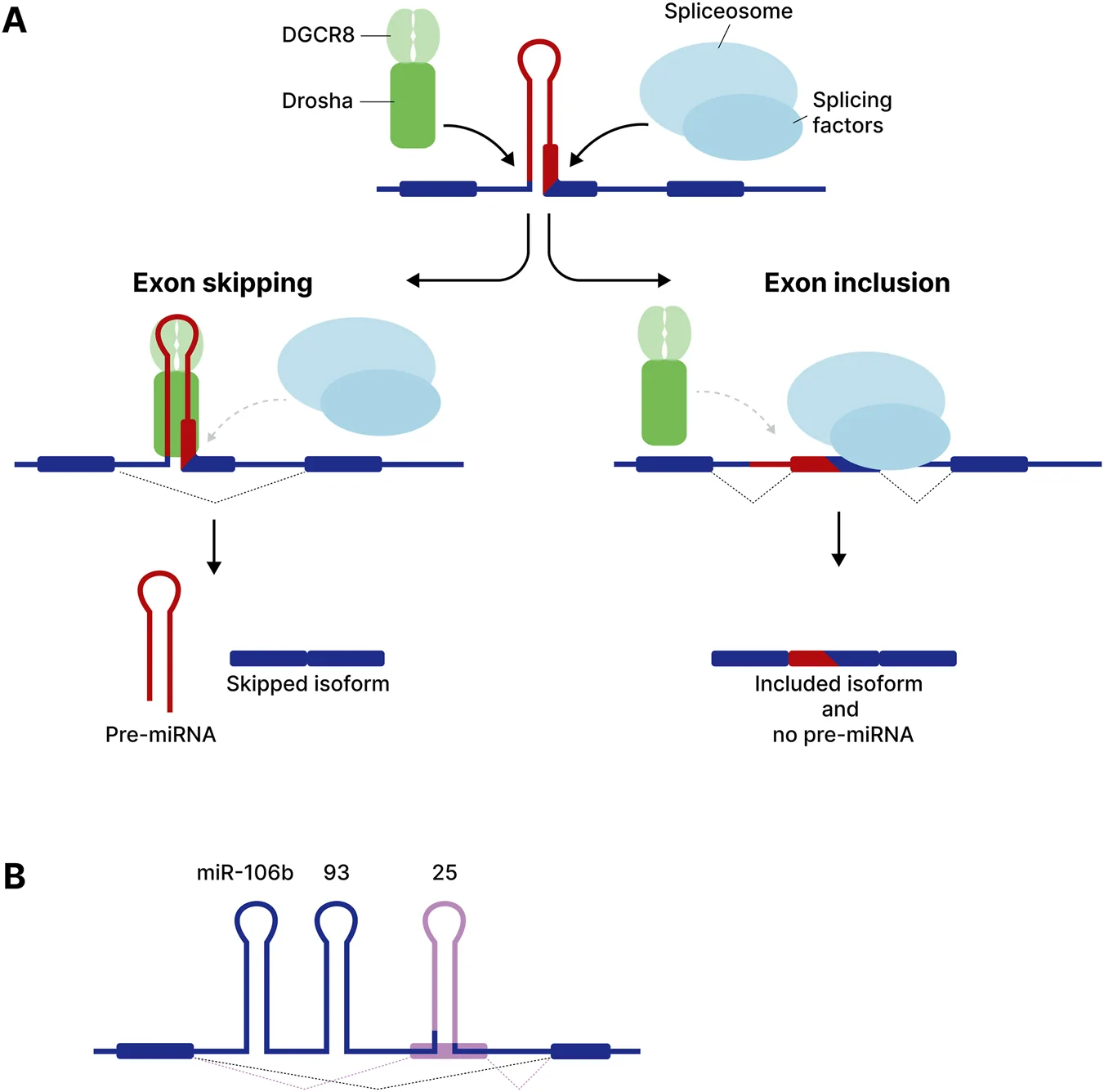
Regulation of miRNA biogenesis through alternative splicing. **(A)** The spliceosome and the Microprocessor complex (comprising one Drosha molecule and two DGCR8 molecules) compete for processing of the same RNA region, leading to two possible outcomes. In the left panel, the pre-miRNA overlaps a splice site, but the splicing machinery does not recognize the internal exon. The Microprocessor complex binds to the RNA transcript in the pre-miRNA region instead of the spliceosomal complex. In this case, both an exon-skipped isoform and pre-miRNA can be generated. In the right panel, the internal exon is recognized by specific splicing factors, causing the pre-miRNA secondary structure to unwind or fail to form. The spliceosome spliced the internal exon before Drosha and DGCR8 can bind, resulting in an isoform containing the alternative exon, while without pre-miRNA production. **(B)** Alternative splicing can also negatively regulate miRNA cluster members located within exons. For example, in the miR-106b~miR-93∼miR-25 cluster, alternative splicing of MCM7 pre-mRNA incorporates the pri-miR-25 sequence into an extended exon 14, reducing its expression.

Clustered miRNAs with precursor sequences overlapping splice sites have also been identified (Melamed et al., 2013; Chang et al., 2015). Melamed et al. (2013) investigated the mouse Mirg gene, which encodes nine miRNAs, including miR-412, whose precursor hairpin overlaps the 3′splice site of an alternatively spliced exon. Splicing at this site and pre-miR-412 processing were found to be mutually exclusive, resulting in a negatively correlation between alternative splicing and miR-412 expression. These findings demonstrate a biological role for alternative splicing in regulating the biogenesis of both individual and clustered miRNAs (Figure 3). Notably, the positioning of miRNAs at exon-intron junctions is not conserved across mammals, even when the miRNAs themselves are conserved (Melamed et al., 2013).

Similarly, if some pre-miRNAs within a cluster are located in intron while others reside entirely within alternatively spliced exon, inclusion of such exon into the mature transcript can reduce the level of the miRNAs encoding within it (Figure 3B). This effect has been demonstrated for the human miR-106b∼miR-93∼miR-25 cluster located in exon 13 of the MCM7 gene. Alternative splicing of the MCM7 pre-mRNA places the pri-miR-25 sequence into an extended exon 14, resulting in reduced miR-25 expression (Agranat-Tamir et al., 2014). Conversely, alternative splicing that relocates the alternative exon into an intron can promote miR-25 production. For miR-24–1 from the miR-23b∼miR-27b∼miR-24–1 cluster, located in intron 15 of the *AOPEP* gene, a hybrid regulatory mechanism has been described. An intragenic promoter produces a transcript comprising three exons flanking intron 15, with pri-miR-24–1 included in exon 4 of this transcript, which is subsequently spliced (Ramalingam et al., 2014). According to Chang et al. (2015), clusters such as miR-130b∼miR-301b and miR-199a-2∼miR-214 also contain pre-miRNAs spanning splice sites.

These finding indicate that alternative splicing can modulate the expression of miRNAs within clusters, either by influencing intron/exon inclusion or through crosstalk between the splicing machinery and the Microprocessor complex.

## 4 Dependence of individual miRNA processing within a polycistronic transcript on neighboring miRNAs

The processing efficiency of pri-miRNAs encoding individual miRNAs is dictated by their intrinsic structural architecture and the presence of specialized sequence motifs that direct and position the Drosha–DGCR8 Microprocessor complex. Canonical pri-miRNA processing determinants comprise a defined set of structural and sequence features: (1) a double-stranded stem of ∼35 base pairs; (2) a single-stranded 3′basal flanking region of at least 9 nt; (3) an apical loop of at least 10 nt; (4) a basal UG motif at the 5′ junction; (5) a flanking CNNC motif in the 3′ basal segment; (6) a bGWG motif; (7) an mGHG motif in the lower stem; and (8) an apical UGUG/UGU motif at the stem–loop junction (Kim et al., 2025). Human pri-miRNA sequences vary considerably in their motif composition; for instance, only 4.5% of human pri-miRNAs harbor the canonical triad of UG, CNNC, and UGUG motifs simultaneously (Kim et al., 2025). Consequently, Microprocessor cleavage efficiency and fidelity (governing isomiR generation) span a wide spectrum across the miRNAome. In general, optimal pri-miRNAs that assemble a full complement of these optimal structural features and motifs (or a greater number of them) are more evolutionarily conserved, undergo more efficient Drosha cleavage, and yield higher steady-state mature miRNA levels than suboptimal precursors that lack key motifs or deviate from canonical structural dimensions.

Specific sequence and structural features of pre-miRNAs that modulate the fidelity and efficiency of Dicer-mediated cleavage have also been identified, as discussed in detail above (see *Introduction*). However, evidence indicates that the final levels of mature miRNAs are largely determined by the kinetics of Drosha processing, with Dicer processing playing a secondary role (Pandey et al., 2022). These principles also apply to the processing of individual miRNAs within polycistronic pri-miRNA transcripts. Within a cluster, precursor hairpins of individual miRNAs may differ in their optimality as substrates for Drosha and Dicer, leading to differential expression of distinct miRNAs within the same cluster.

Notably, the processing of a given miRNA within a polycistronic transcript can be influenced by the processing kinetics of neighboring miRNAs. Specifically, the presence of structurally and sequence-optimized pri-miRNAs within a polycistronic transcript can facilitate the processing of suboptimal cluster members, a phenomenon termed “cluster assistance” (Figure 4). For example, in the miR-11∼miR-998 cluster, expression of miR-998 is entirely dependent on the presence of the neighboring miR-11 gene within the same primary transcript, as demonstrated in experiments on *Drosophila* S2R + cells (Truscott et al., 2016). In the absence of pri-miR-11, processing of pri-miR-998 fails. This dependency arises because pri-miR-998 has an unusually long precursor (142 nt versus the average 94 nt), rendering it a poor substrate for the Microprocessor. The presence of a neighboring optimal precursor hairpin ensures proper the whole pri-miRNA architecture and enables efficient processing. It is also noteworthy that miR-11 facilitates the processing of miR-998, regardless of whether it is located upstream or downstream of miR-998 in the miR-11∼miR-998 gene (Truscott et al., 2016).

**FIGURE 4:**
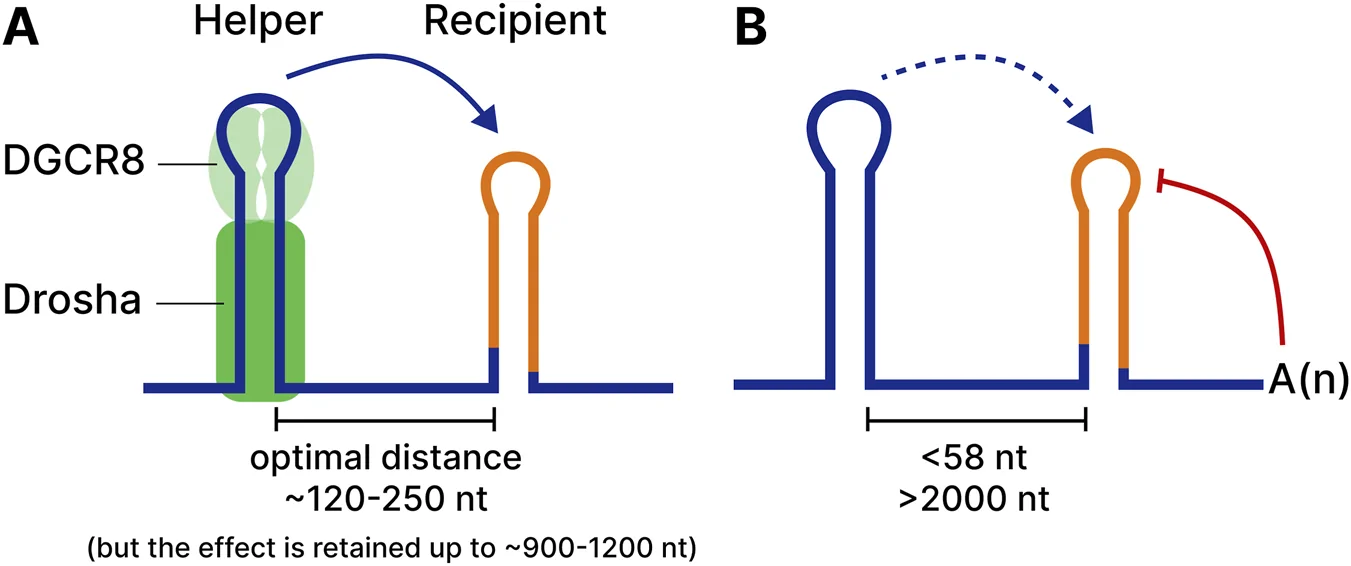
Principle of cluster assistance. **(A)** The presence of a helper hairpin with an optimal structure within the primary transcript enhances Microprocessor (Drosha–DGCR8)-mediated processing of a recipient hairpin with suboptimal structural or sequence features. **(B)** Factors that limit cluster assistance. Inter-hairpin distances exceeding ∼2,000 nt or falling below ∼58 nt, as well as proximal polyadenylation (A(n)) site, can abolish or substantially weaken cluster assistance. According to (Shang et al., 2020), the optimal distance between helper and recipient hairpins is approximately 120–250 nt; however, cluster assistance can still be partially retained at distances of ∼900–1,200 nt (Fang and Bartel, 2020; Hutter et al., 2020; Shang et al., 2020; Jang et al., 2025).

An additional example is the non-canonical (Dicer-independent) precursor hairpin of miR-451. This hairpin is a weak substrate for the Microprocessor due to its short loop (only 4 nt) and short stem (31 bp instead of the optimal ∼35 bp). Efficient processing of pri-miR-451 requires its inclusion in a cluster with the canonical pri-miR-144, which recruits the Microprocessor and locally transfers it to pri-miR-451, as demonstrated in human leukemia K562 cells (Shang et al., 2020).

The dependence of processing efficiency on neighboring miRNAs has also been experimentally confirmed in HEK293T cells for miR-374a, miR-374b, and miR-449c, whose precursor hairpins possess short terminal loops (Fang and Bartel, 2020; Shang et al., 2020). Interestingly, lengthening their loops restores processing independently of neighboring miRNAs (Shang et al., 2020).

A similar mechanism has been observed for the miR-100∼let-7∼miR-125 cluster in *Drosophila* S2R+ and Kc167 cell lines (Truscott et al., 2016; Pandey et al., 2022), in which miR-125 expression depends on the processing of the miR-100 and let-7 precursor hairpins. The absence of pri-let-7 in this polycistronic transcript reduces processing of both pri-miR-100 and pri-miR-125. Furthermore, (Pandey et al., 2022), using chimeric variants of the conserved miR-100∼let-7∼miR-125 cluster, demonstrated that the position and order of miRNAs within the cluster critically influence processing. Altering the relative order of precursor hairpins can either enhance or attenuate the biogenesis of individual miRNAs. This spatial sensitivity points to intricate structural hierarchies within primary transcripts and implies that the linear organization of hairpins within a cluster is actively shaped by evolutionary selection. One possible explanation for the importance of cluster member order is that miRNA processing within a cluster occurs sequentially in the 5′→3′direction, as demonstrated, for example, in the Epstein-Barr virus BHRF1 miRNA cluster (Haar et al., 2016). However, as noted above for the miR-11∼miR-998 cluster, positioning the helper hairpin upstream of the suboptimal recipient is not an absolute prerequisite for efficient processing. Similar findings were reported for cluster assistance in the miR-144∼miR-451 cluster (Shang and Lai, 2023). Thus, the proposed rule of sequential 5′→3′processing does not appear to apply strictly to all miRNA clusters; alternatively, it may be bypassed in the presence of additional factors, as discussed below. Notably, the helper hairpin must be an optimal substrate for the Microprocessor but does not necessarily need to be efficiently cleaved by Dicer during pre-miRNA processing (Shang and Lai, 2023).

Interestingly, miRNA clusters are often enriched in miRNAs with suboptimal precursor hairpins (Shang et al., 2020). Clustering is thought to increase the local concentration of the Drosha/DGCR8 Microprocessor, facilitating recognition of suboptimal substrates when they are in close proximity (Shang et al., 2020; Vilimova and Pfeffer, 2023). Within clusters, such miRNAs are expressed whose precursor hairpins are either not processed at all outside the cluster or are processed with very low efficiency. This may help explain the prevalence of miRNA clusters in the genome.

In clusters with two or more members, the proximity of a suboptimal pri-miRNA to an optimal pri-miRNA confers a competitive advantage by stimulating its biogenesis (Shang et al., 2020). Increasing the distance between the optimal and suboptimal pri-miRNA to 2,000–3,072 nucleotides abolishes or substantially weakens the cluster assistance effect (Figure 4) (Fang and Bartel, 2020; Shang et al., 2020). According to (Shang et al., 2020), the optimal distance between helper and recipient hairpins is approximately 120–250 nt; nevertheless, cluster assistance remains detectable at distances of ∼900–1,200 nt assistance (Fang and Bartel, 2020; Hutter et al., 2020; Shang et al., 2020; Jang et al., 2025). Inter-hairpin distances exceeding 2000 nt may reduce the efficiency of Microprocessor transfer from the helper hairpin to the recipient hairpin, potentially because the local concentration of the complex decreases over longer distances. In autonomous miRNA clusters coupling between transcription and processing may provide an additional explanation: when the hairpins are separated by a substantial distance, the optimal helper hairpin may be processed before transcription of the downstream suboptimal recipient hairpin is completed, preventing cooperative Microprocessor recruitment. However, these hypotheses remain to be experimentally verified. Conversely, excessively short distances between cluster members also reduce the processing efficiency of suboptimal pri-miRNA. For example, in the Epstein-Barr virus BHRF1 cluster, increasing the 58-nucleotide spacer between pri-miR-BHRF1-2 and −3 improved processing of the suboptimal pri-miR-BHRF1-3 (Haar et al., 2016). In the miR-144∼miR-451 cluster, processing of the suboptimal pri-miR-451 was most efficient with spacer lengths of 120 nt (the endogenous distance) and 250 nt between the hairpins, while a short 30-nt spacer abolished miR-451 maturation (Shang et al., 2020). Evidently, such a short spacer does not provide sufficient flanking single-stranded sequence for proper recognition of pri-miR-451 by the Microprocessor complex after cleavage of pri-miR-144. The effectiveness of cluster assistance is also limited by proximity to a polyadenylation site (poly(A) site) (Haar et al., 2016). This could indicate coupling between pri-miRNA processing and transcription, such that an overly short distance to the poly(A) site hinders the timely recognition of the precursor hairpin structure within the nascent transcript.

The viral mir-K10/12 cluster, expressed in human BCBL-1 lymphoma cells by Kaposi’s sarcoma-associated herpesvirus (KSHV) and encoding 10 miRNA precursors, provides an example showing that cluster assistance is not restricted to the nearest hairpins or those that are suboptimally folded (Vilimova et al., 2021). In this cluster, the presence of pri-miR K1 and pri-miR K3 determines the expression of all other miRNAs in the cluster. Replacement of pri-miR K1 with a heterologous pri-let-7a-1 with an optimal structure preserves expression of the entire cluster at wild-type levels. Thus, the presence of a pri-miRNA with a canonical structure at this position, rather than its unique sequence, is critical. Deletion of pri-miR K1 and pri-miR K3 disrupts global folding of the transcript. The importance of tertiary transcript structure has also been demonstrated in the murine bicistronic miR-497∼miR-195 cluster (Lataniotis et al., 2017). These observations highlight the significance of local context and secondary/tertiary transcript structure, as well as indicate that regulatory mechanisms extend beyond simple recruitment of the Drosha/DGCR8 Microprocessor and facilitation of its “reloading” onto adjacent hairpins.

Several studies (Chul Kwon et al., 2020; Fang and Bartel, 2020) have demonstrated that cluster assistance in the miR-144∼miR-451 cluster requires the protein ERH (enhancer of rudimentary homolog), which interacts with the Microprocessor (Chul Kwon et al., 2020), as well as the scaffold attachment factor B proteins, SAFB1 and SAFB2 (Drakouli et al., 2017). Loss of ERH abolishes stimulation of suboptimal miR-451 hairpin processing, while the processing of miR-144 remains unaffected. Chul Kwon et al. (2020) further established that ERH act as a third stable Microprocessor component alongside Drosha and DGCR8 and influences the processing of other clustered miRNAs with suboptimal precursor hairpins, such as miR-181b-1 and miR-425.

SAFB2 is another protein that facilitates cluster assistance (Hutter et al., 2020). In the bicistronic miR-15a∼miR-16–1 cluster, pri-miR-15a is a suboptimal Drosha substrate due to a large unpaired region at the base of the stem. Efficient processing requires the presence of pri-miR-16–1 and SAFB2, which binds Drosha and acts as a necessary cofactor (Hutter et al., 2020). SAFB2 knockdown in two different cell lines reduced expression of eight additional clustered miRNAs, including miR-181b, miR-92a, and miR-425, indicating its broader role in cluster assistance. ERH also contributes to pri-miR-15a processing, and SAFB1, which is highly homologous to SAFB2, can partially substitute for SAFB2 (Hutter et al., 2020). Interestingly, in the bicistronic transcript of the miR-15a∼miR-16–1 cluster, the optimal pri-miR-16–1 hairpin is located downstream of the suboptimal pri-miR-15a, suggesting that RBPs may “override” the 5′→3′sequential processing rule of polycistronic transcripts.

Two models have been proposed for ERH and SAFB function in cluster assistance (Fang and Bartel, 2020). The first model involves Microprocessor dimerization via ERH and SAFB with one complex binding to the “helper” hairpin and the second complex binding to the “recipient” hairpin. The second model suggests that the Microprocessor is retained after cleavage of the helper hairpin, allowing it to subsequently recognize and process the neighboring hairpin; the precise mechanistic roles of ERH and SAFB in this scenario was not specified. A recent study by Jang et al. provides deeper mechanistic insight into the dedicated functions of ERH and SAFB proteins during cluster cooperation (Figure 5), highlighting the primary role of the Microprocessor retention and handover mechanism (Jang et al., 2025). Specifically, the authors demonstrated that ERH and SAFB2 perform distinct, non-redundant roles: ERH facilitates the processing of both clustered and monocistronic pri-miRNAs, whereas SAFB2 is predominantly required for the processing of suboptimal hairpin structures within clusters. The dependence on ERH is dictated primarily by the suboptimal nature of the pri-miRNA, rather than by its cluster organization, flanking sequences, or the presence of a helper hairpin. Hence, ERH acts as a general cofactor that boosts the processing of suboptimal pri-miRNAs. Mechanistically, using cellular and lysate-based systems, it was shown that ERH binds to DGCR8 and enhances Microprocessor activity by stabilizing it in a more active conformation. The authors further note that, because this effect could not be recapitulated in a purified recombinant system, additional cofactors are likely required for ERH function. In contrast, SAFB2 is not required for processing of individual suboptimal pri-miRNAs, but is essential for cluster assistance. Experimental evidence from Jang and colleagues (Jang et al., 2025) suggests that SAFB2 facilitates transfer of the Microprocessor from the helper to the recipient hairpin, likely through SAFB2 multimerization, a property previously demonstrated for this protein (Hutter et al., 2020). This mechanism does not appear to involve bringing multiple Microprocessor complexes into close proximity (Jang et al., 2025). These findings therefore argue against the first model proposed in (Fang and Bartel, 2020), which postulates Microprocessor dimerization. By interacting with both the Microprocessor and RNA, SAFB2 may retain the complex in the vicinity of the recipient hairpin. However, whether additional cofactors participate in this process remains unresolved. It is plausible that SAFB2 multimerization contributes to the formation of a local ribonucleoprotein (RNP) microenvironment (conceptually analogous to the concentrated phase-separated hubs formed at super-enhancers), thereby increasing the local concentration of all components required for pri-miRNA processing. This could also account for the decreased processing efficiency of suboptimal pri-miRNAs when the inter-hairpin distance exceeds 2000 nt, as the local effective concentration may drop substantially over such distances.

**FIGURE 5:**
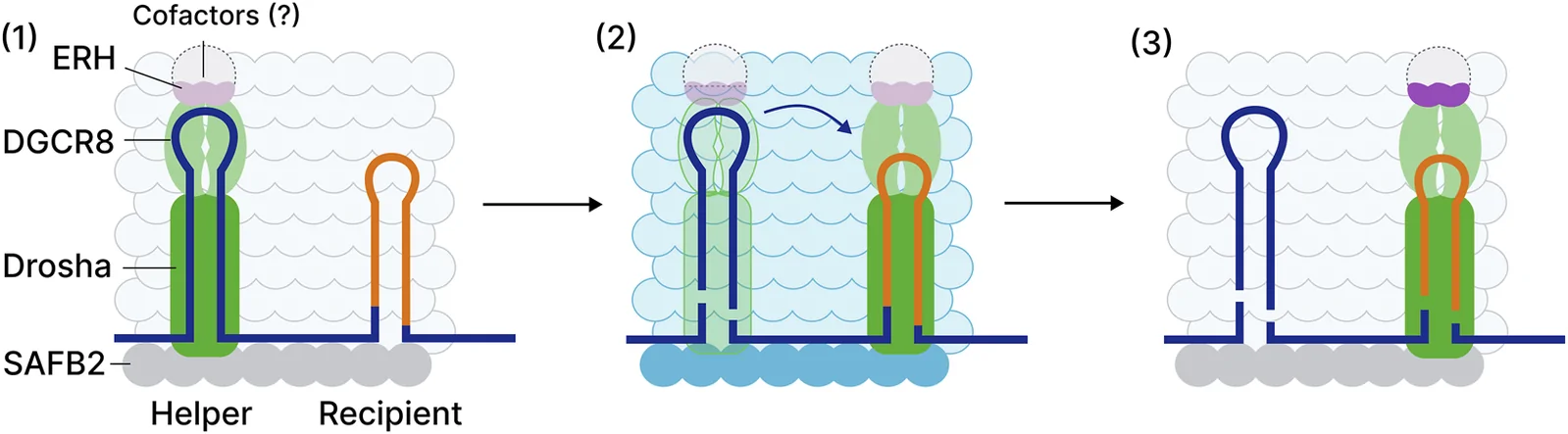
Mechanistic framework for ERH- and SAFB2-mediated cluster assistance. (1) The optimal helper hairpin recruits Microprocessor for primary processing. (2) SAFB2 mediates the tethering and handover of the Microprocessor to the neighboring recipient hairpin via multimerization. (3) ERH boosts the processing efficiency of the suboptimal recipient hairpin in cooperation with yet-unidentified cellular cofactors (Jang et al., 2025). The exact structural basis of SAFB2 function and the full complement of interacting factors warrant further investigation.

Synthesis of available evidence suggests that cluster assistance operates through two not fully overlapping mechanistic modes. The first is an intrinsic *cis*-structural mode, wherein suboptimal hairpin processing is governed solely by the structural and spatial topology of the polycistronic transcript including: inter-hairpin spacing, linear order, higher-order tertiary architecture, and proximity to the polyadenylation signal, without an obligatory requirement for specialized auxiliary proteins. The second is an extrinsic protein-mediated mode, wherein dedicated cofactors (such as ERH and SAFB1/2) physically engage the Microprocessor and transcript scaffold. This protein-assisted mechanism facilitates complex retention and handover, thereby licensing cluster assistance independent of the relative linear orientation of the helper and recipient hairpins.

RNA polymerase II (Pol II) elongation dynamics provide another regulatory layer that modulates Drosha processing of suboptimal clustered miRNAs. In *Drosophila*, the Microprocessor subunit Pasha (DGCR8) binds the C-terminal domain (CTD) of the Pol II subunit Rpb1 through a conserved WW domain, requiring Cdk9-mediated CTD phosphorylation (Church et al., 2017). Disrupting Cdk9 activity impairs Pasha recruitment to elongating Pol II and alters global processing stoichiometry: cleavage of pri-miRNAs containing an apical UGU motif is promoted, whereas processing of precursors lacking this motif is suppressed. Consequently, Pol II CTD phosphorylation acts as a licensing mechanism for the co-transcriptional processing of suboptimal, UGU-lacking pri-miRNAs, whereas canonical UGU-containing precursors are efficiently excised independently of direct physical association between the Microprocessor and Pol II. Because pri-miRNA processing can occur both co-transcriptionally and post-transcriptionally (PMID: 28250203), and ERH promotes the processing of suboptimal hairpins independently of the Pol II transcriptional machinery (Fang and Bartel, 2020; Jang et al., 2025), the transcription-coupled and ERH/SAFB2-dependent mechanisms may function independently, or only partially depend on one another. Nevertheless, Pol II may enhance the efficiency of ERH-mediated processing (Fang and Bartel, 2020).

Beyond processing kinetics, the final stoichiometric ratio of mature miRNAs is dictated also by cleavage fidelity. Positional variability during Drosha and, especially, Dicer cleavage generates template-dependent 5′-isomiRs bearing shifted seed sequences (Lee et al., 2023; Le et al., 2024; Maltseva et al., 2024). Because even a single-nucleotide shift at the 5′ terminus dramatically alters target mRNA recognition, these isoforms engage a distinct regulatory repertoire. Consequently, the generation of alternative isoforms directly depletes the canonical miRNA pool and variations in cleavage precision represent a significant post-transcriptional mechanism driving expression divergence among cotranscribed cluster members.

## 5 RNA-binding proteins in the regulation of miRNA biogenesis within clusters

RNA-binding proteins (RBPs) provide an additional layer of regulation by recognizing specific sequences in miRNA precursors and modulating their processing in various biological contexts (Table 1). For instance, beyond its canonical roles in mRNA metabolism (including alternative splicing, nuclear export, stability, and translation), heterogeneous nuclear ribonucleoprotein A1 (hnRNP A1) directly regulates microRNA biogenesis, particularly within polycistronic loci (Feng et al., 2022). A classic example is its interaction with the miR-17–92 cluster, where hnRNP A1 binds the pri-miR-18a and promotes Drosha-mediated cleavage in HeLa cells (Guil and Cáceres, 2007). Specifically, hnRNP A1 binds to the terminal loop of pri-mir-18a, inducing allosteric destabilization of stem base-pairing, thereby facilitating cleavage by Microprocessor (Kooshapur et al., 2018). Conversely, in the let-7a-1∼let-7f-1∼let-7d cluster, hnRNP A1 negatively regulates pri-let-7a-1 processing by binding its conserved terminal loop and suppressing cleavage by Drosha in *in vitro* system (Michlewski and Cáceres, 2010). Intriguingly, although hnRNP A1 binds to the homologous topological domain (the terminal loop) in both pri-miR-18a and pri-let-7a-1, it exerts opposing regulatory outcomes on their maturation: promoting Drosha cleavage of pri-miR-18a while repressing pri-let-7a-1 processing. This divergence indicates that the functional outcome of hnRNP A1 engagement is governed not merely by binding-site location, but by the unique structural geometry and conformational dynamics of the individual hairpin. Furthermore, hnRNP A1 can be recruited to transcripts harboring N6-метиладенозин (m6A), functioning as an indirect or direct reader of this epitranscriptomic mark (Kumar et al., 2022). Because m6A deposition can directly induce local RNA structural remodeling (Wang X. et al., 2025), it remains a compelling open question whether the context-dependent effects of hnRNP A1 on pri-miRNA processing are modulated by the local m6A modification status within or near its binding site.

**TABLE 1:** Validated interactions of RNA-binding proteins with clustered miRNA precursors.

RNA-binding protein	miRNA precursor/miRNA	Effect of interaction*	Cell type	References
BUD13	pri-miR-144	Stabilization of the primary transcript	K562, *in vitro*	(Nussbacher and Yeo, 2018)
C9ORF114	pri-miR-145	Stimulates miR-145 maturation	HEPG2 In these cell lines only binding was demonstrated: DLD-1, DU145, MCF7, NTERA-2	(Treiber et al., 2017)
DDX3X	pri-/pre-miR-20a	Stabilization of the cluster primary transcript	K562, U2OS	(Zhao et al., 2016; Nussbacher and Yeo, 2018)
DDX3X	pri-miR-145	Stimulates miR-145 maturation	U2OS	(Zhao et al., 2016)
DDX3X	pri-miR-19b	Stimulates miR-19b maturation	U2OS	(Zhao et al., 2016)
DZIP3	pre-miR-20a	n.d	DLD-1, DU145, MCF7	(Treiber et al., 2017)
hnRNP A1	pri-let-7a-1	Inhibition of processing by Drosha	Demonstrated *in vitro*	(Michlewski and Cáceres, 2010)
hnRNP A1	pri-miR-18a	Stimulation of processing by Drosha	HeLa	(Guil and Cáceres, 2007; Kooshapur et al., 2018)
hnRNP A1	pri-miR-18a	n.d	A549, DLD-1, DU145, HEPG2, Jurkat, LN-229, MCF7, NTERA-2, Raji, SK-MEL-28, SK-N-MC	(Treiber et al., 2017)
hnRNP A1	pri-miR-143	n.d	DLD-1, DU145, HEPG2 Jurkat, LN-229, MCF7, NTERA-2, SK-MEL-28, SK-N-MC	(Treiber et al., 2017)
ILF3	pri-miR-144	Stabilization of the primary transcript	K562, *in vitro*	(Nussbacher and Yeo, 2018)
KSRP (KHSRP)	pri-let-7a-1	Promotes let-7a-1 maturation	Demonstrated *in vitro*	(Michlewski and Cáceres, 2010)
KSRP (KHSRP) sumoylated	pri-let-7 family	Inhibition of processing by Drosha	DU145	(Yuan et al., 2017)
Lin28**	pre-let-7 family	Recruits terminal uridylyltransferases TUT4, blocks Dicer-mediated processing, and promotes degradation	HEK293T, Huh7, embryonic stem cells and embryocarcinoma cells	(Heo et al., 2008; 2009; Rybak et al., 2008; Dou et al., 2020)
Lin28	pre-miR-143	Recruits terminal uridylyltransferase TUT4, prevents maturation, and promotes degradation	HEK293T	(Heo et al., 2009)
Lin28	pre-miR-200c	Recruits terminal uridylyltransferase TUT4, prevents maturation, and promotes degradation	Demonstrated *in vitro*	(Heo et al., 2009)
MATR3	pre-miR-29b-2	n.d	DLD-1, DU145, HEPG2, LN-229, MCF7, NTERA-2, SK-MEL-28, SK-N-MC	(Treiber et al., 2017)
PUM1	pri-miR-199a-1	Stimulates miR-199a maturation	HEK293, SK-N-MC	(Treiber et al., 2017)
PUM2	pri-miR-199a-2	Stimulates miR-199a maturation	HEK293, SK-N-MC	(Treiber et al., 2017)
PTBP1	pre-miR-29b-2	n.d	A549, DLD-1, DU145, HEPG2, Jurkat, LN-229, MCF7, NTERA-2, Raji, SK-MEL-28, SK-N-MC	(Treiber et al., 2017)
PTBP3	pre-miR-29b-2	n.d	A549, DLD-1, DU145, HEPG2, Jurkat, LN-229, MCF7, NTERA-2, Raji, SK-MEL-28, SK-N-MC	(Treiber et al., 2017)
QKI	miR-20a	Stabilizes mature miR-20a	LN18 and U343 cells	(Chen et al., 2012)
Rbfox2	pri-miR-20b	Alters the conformation of the pri-miRNA hairpin, inhibits its recognition and cleavage by Drosha	SHSY-5Y, и MCF7, HeLa	(Chen et al., 2016)
RBM10	pre-let-7g	n.d	A549, DLD-1, DU145, HEPG2, Jurkat, LN-229, MCF7, NTERA-2, Raji, SK-MEL-28, SK-N-MC	(Treiber et al., 2017)
SAFB2	Primary transcript of the miR-15a∼16–1 cluster	Stimulates cluster assistance and processing of pri-miR-15a		(Hutter et al., 2020)
SART3	pre-miR-18b	n.d	A549, DLD-1, DU145, HEPG2, LN-229, MCF7, SK-MEL-28	(Treiber et al., 2017)
SRSF3	Primary transcript of the miR-17–92 cluster	Promotes selective processing of pri-miR-17 and pri-miR-20a	LIM1215, HEK293T	(Ratnadiwakara et al., 2023)
SRSF3	pri-miR-16	Promotes selective processing by Drosha	Demonstrated *in vitro*	(Auyeung et al., 2013)
SRSF3	pri-miR-30a	Promotes selective processing by Drosha	Demonstrated *in vitro*	(Kim et al., 2018)
SRSF3	pri-miR-30c	Promotes selective processing by Drosha	Demonstrated *in vitro*	(Fernandez et al., 2017)
SRSF3	pri-miR-142	Promotes selective processing by Drosha	Demonstrated *in vitro*	(Kim et al., 2018)
TDP-43	pri-miR-132, pri-miR-143/pre-miR-143	Nuclear TDP-43 facilitates processing of pri-miR-132 and pri-miR-143; cytoplasmic TDP-43 facilitates processing of pre-miR-143	HEK293T, SH-SY5Y	(Kawahara and Mieda-Sato, 2012)
TRIM26	pre-miR-18b	n.d	DLD-1, HEPG2, MCF7	(Treiber et al., 2017)
TRIM71 (LIN41)	pri-miR-29a/pre-miR-29a	Depletion of TRIM71 leads to reduced mature miR-29a levels	NTERA-2, HEPG2 (only bindind)	(Treiber et al., 2017)
TRIM71 (LIN41)	pre-miR-100	n.d	NTERA-2	(Treiber et al., 2017)
TRIM71 (LIN41)	pre-miR-1-2	n.d	NTERA-2, HEPG2	(Treiber et al., 2017)
YB-1 (YBX1)	pri-miR-29b-2/pre-miR-29b-2	Impedes recruitment of Drosha and Dicer	HeLa In these cell lines only binding was demonstrated: A549, DLD-1, DU145, HEPG2, Jurkat, LN-229, MCF7, NTERA-2, Raji, SK-MEL-28, SK-N-MC	(Wu et al., 2015; Treiber et al., 2017)
YBX2	pre-miR-29b-2	n.d	DU145, MCF7	(Treiber et al., 2017)
YBX3	pre-miR-29b-2	n.d	DLD-1, DU145, HEPG2, Jurkat, LN-229, MCF7, NTERA-2, Raji, SK-N-MC	(Treiber et al., 2017)
ZC3H10	pri-miR-143	Inhibition of processing by Drosha	NTERA-2, SK-N-MC	(Treiber et al., 2017)
ZC3H7A	pre-miR-16–2	n.d	A549, HEPG2, LN-229, MCF7	(Treiber et al., 2017)
ZC3H7A	pre-miR-29a	n.d	A549, HEPG2, LN-229	(Treiber et al., 2017)
ZC3H7B	pre-miR-16–2	n.d	HEPG2, LN-229, MCF7	(Treiber et al., 2017)
ZC3H7B	pre-miR-29a (weak)	n.d	A549, DLD-1, HEPG2, MCF7, SK-MEL-28	(Treiber et al., 2017)

* n.d. (no data) indicates that binding of the RBP, to the miRNA, precursor has been shown, but its functional significance for processing has not been established.

** Sumoylation of Lin28A at lysine 15 (K15) significantly enhances its affinity for binding to pre-let-7. This modification effectively blocks pre-let-7, processing, thereby suppressing the production of mature let-7 miRNAs (Dou et al., 2020).

KSRP (KHSRP), another RBP, binds to the same terminal loop region as hnRNP A1 and acts as a positive regulator of let-7a-1 maturation. The balance between the repressor hnRNP A1 and the activator KSRP determines the final let-7a level in differentiated human cells (Michlewski and Cáceres, 2010). Interestingly, sumoylation of KSRP converts it into a negative regulator, inhibiting processing of let-7 family pri-miRNAs (Yuan et al., 2017). These findings highlight that post-translational modifications can modulate the activity of RBPs in cluster-specific miRNA regulation. In our view, the findings for KSRP illustrate a broader regulatory principle highlighted throughout this review: the magnitude and directionality of an RBP’s effect on miRNA maturation are governed not only by protein identity and binding-motif topology, but also by its post-translational modification (PTM) state. As discussed below, for example, phosphorylation of hnRNP A2/B1 enhances both its protein stability and its competence as an m6A reader, thereby selectively promoting the maturation of pri-miR-93. Thus, signal-responsive PTMs on RBPs establish an agile, dynamic layer of post-transcriptional control that enables cells to rapidly remodel the stoichiometric output of polycistronic miRNA clusters in response to microenvironmental cues.

The protein TDP-43 is involved in the post-transcriptional regulation of the biogenesis of specific miRNAs both in the nucleus and in the cytoplasm (Kawahara and Mieda-Sato, 2012). In the nucleus, TDP-43 functions as a component of the Drosha-DGCR8 complex and directly binds a specific subset of pri-miRNAs, including the clustered pri-miR-132 and pri-miR-143, thereby facilitating their processing. In the cytoplasm, TDP-43 directly interacts with Dicer (in a complex with TRBP) and with the terminal loops of certain pre-miRNAs, including clustered pre-miR-143, promoting their further cleavage into mature miRNAs. It has been shown that miR-132 is indispensable for neurite outgrowth in Neuro2a cells. Whereas depletion of TDP-43 impairs neurite outgrowth, ectopic overexpression of pri-miR-132 fully rescues this defect (Kawahara and Mieda-Sato, 2012). In neurodegenerative disorders such as amyotrophic lateral sclerosis (ALS) and frontotemporal lobar degeneration (FTLD), pathological mutations and the abnormal accumulation of TDP-43 into cytoplasmic aggregates compromise its physiological RNA-processing functions. Consequently, the neurodegenerative cascades associated with TDP-43 loss-of-function proteinopathies may stem, in part, from impaired miR-132 biogenesis.

The protein YB-1 (Y box-binding protein 1), primarily known as an mRNA packaging protein, has also been identified as a modulator of miRNA processing (Wu et al., 2015). Specifically, YB-1 binds to the precursor of the tumor suppressor miR-29b-2 (part of the miR-29c∼miR-29b-2 cluster) and blocks recruitment of both the Microprocessor complex (Drosha/DGCR8) and Dicer. Overexpression of YB-1 in human U251-MG glioblastoma cells stimulates proliferation, including through suppression of miR-29b-2 maturation.

Polycistronic pri-miRNAs present additional challenges for the Microprocessor complex, as long transcripts can fold into complex structures. For instance, the miR-17–92 cluster transcript folds into a compact globular structure *in vitro*, occluding some Drosha cleavage sites and reducing Microprocessor accessibility (Ratnadiwakara et al., 2023). Binding of SRSF3 to CNNC motifs modifies the global structure of the entire transcript, selectively promoting processing of pri-miR-17 and pri-miR-20a in human colorectal LIM1215 cells (Ratnadiwakara et al., 2023). Overexpression of SRSF3 enhances proliferation of LIM1215 cells through miR-17a/20a-mediated suppression of the cell cycle inhibitor CDKN1A expression. SRSF3 binding 17–18 nt downstream of Drosha cleavage site also enhances cleavage of other clustered pri-miRNAs *in vitro*, including pri-miR-16 (Auyeung et al., 2013), pri-miR-30a (Kim et al., 2018), pri-miR-30c (Fernandez et al., 2017), and pri-miR-142 (Kim et al., 2018).

Rbfox2, in addition to its well-known role in splicing, directly regulates the biogenesis of certain miRNAs by binding to the conserved GCAUG motif in their precursors (Chen et al., 2016). This interaction alters pri-miRNA hairpin conformation, preventing proper recognition and cleavage by Drosha, thereby reducing mature miRNA levels, including the clustered miRNA miR-20b in SHSY-5Y and MCF7 cell lines. Decreased Rbfox2 and increased mature miR-20b are associated with increased levels of the tumor suppressor protein PTEN in MCF7 cells. Sequence analysis indicates that over 120 miRNA precursors contain the Rbfox recognition motif, suggesting that a substantial subset of miRNAs may be regulated by this protein family (Chen et al., 2016).

Fragile X-related protein 1 (FXR1) is an RNA-binding protein (RBP) that regulates multiple stages of post-transcriptional gene expression, including miRNA processing, mRNA stability, and translation. It has also been implicated in miRNA-mediated gene silencing through its interactions with components of the RISC (Khan et al., 2024a). Dysregulated FXR1 expression has been reported in diverse malignancies, including lung, ovarian, hepatocellular, prostate, glioblastoma, esophageal, and colorectal cancers (Khan et al., 2024a; 2024b). FXR1 is required for the efficient maturation of certain neuronal miRNAs, as it can form complexes with Dicer and specific pre-miRNAs. In addition, FXR1 may enhance RISC-mediated translational repression of particular target mRNAs through direct interactions with the miRNA machinery (Khan et al., 2024a). There is evidence suggesting an interaction between FXR1 and the miRISC complex containing clustered miR-219, miR-23b, and miR-200b, which are important for eye and cranial cartilage development in *Xenopus laevis* (Gessert et al., 2010). Notably, FXR1 activity is context dependent. In addition to promoting miRNA/RISC-associated translational repression, FXR1 can stabilize selected mRNAs and, under particular cellular conditions, promote their translation. Thus, its effect depends on the target transcript, associated miRNA or RBP partners, cellular context, and possibly the specific FXR1 isoform involved (Khan et al., 2024a).

Treiber et al. (2017), performed pull-down assays using immobilized *in vitro* RNA hairpins and lysates from 11 cell lines, identifying 180 RBPs that specifically bind to precursors of one or more miRNAs (72 precursors in total). Of these, interactions with 26 proteins were validated. Table 1 summarizes the validated RBP interactions with clustered miRNA precursors. These proteins recognize specific sequences either in the loop or stem regions of RNA hairpins, and in some cases, combined recognition of both regions has been observed (e.g., TRIM26). Knockdown or knockout of several of these RBPs demonstrated that their depletion can either increases or decreases mature miRNA levels (Treiber et al., 2017), indicating that they function as inhibitors or stimulators of precursor hairpin processing (Table 1).

Nussbacher and Yeo (2018) used eCLIP (enhanced CrossLinking and Immunoprecipitation) to analyze 126 RBPs and found that 116 directly bind at least one miRNA precursor. Most of these RBPs interact with fewer than 25 unique precursors, and approximately 68% of RBP–miRNA precursor interactions are cell-type-specific, highlighting the importance of context-dependent post-transcriptional regulation.

Because the cluster-assistance cofactor ERH lacks intrinsic RNA-binding activity, and its stimulation of suboptimal hairpin processing appears to require auxiliary cellular factors (Jang et al., 2025), sequence-specific RNA-binding proteins (RBPs) may serve as critical recruiting adaptors that confer substrate selectivity to ERH. Consequently, determining how specific RBPs functionalize or depend on ERH during pri-miRNA processing represents an important avenue for future mechanistic dissection.

In summary, RBPs regulate miRNA expression across multiple biogenesis stages: stabilizing primary transcripts, promoting or inhibiting the cleavage of pri- and pre-miRNA precursors, and governing precursor decay (Table 1). The net regulatory effect of an RBP on individual clustered miRNAs is dictated by at least three independent parameters: (1) the binding site within the precursor hairpin, which determines whether access of Drosha/Dicer is facilitated or blocked; (2) the post-translational status of the RBP itself (phosphorylation, SUMOylation), which can enhance or even invert the direction of the effect of the same protein; and (3) the cell-type-specific profile of auxiliary cofactors and competing RBPs, due to which an interaction validated in one system may not be reproduced in another. Notably, the cellular availability of RBPs may also be regulated by circular RNAs (circRNAs), which can act as protein sponges or decoys, thereby adding another layer of post-transcriptional regulation (Khan et al., 2022). Such multifactoriality may explain why a single RBP (such as hnRNP A1) can stimulate the processing of one miRNA precursor but suppress processing of another. By integrating these regulatory layers, cells achieve agile, post-transcriptional fine-tuning of individual miRNA levels from a single polycistronic transcript.

Two additional aspects merit consideration. Although they do not directly explain differential expression of miRNAs produced from a polycistronic transcript, they may contribute to differences in the functional activity of individual cluster-encoded miRNAs, including their capacity to repress target mRNAs. First, the pool of miRNAs available for loading into RISC can be modulated by circular RNAs (circRNAs), which may function as endogenous miRNA sponges (Khan et al., 2022). For example, circRNAs that interact with clustered miR-203a-3p, miR-296, miR-214, miR-17, miR-29a-3p, and miR-145 have been identified (Khan et al., 2022). Second, individual miRNAs can differ markedly (by more than 100-fold) in their extent of association with Ago proteins, which may further influence their effective incorporation into RISC and, consequently, the efficiency of target-mRNA repression (Flores et al., 2014; Kingston and Bartel, 2019). Consequently, equimolar miRNA abundance does not guarantee equivalent levels of functional miRNA-loaded RISC (miRISC). Indeed, the cellular abundance of miRISC complexes serves as a markedly better predictor of miRNA repressive potency than total cellular miRNA expression (Flores et al., 2014). Furthermore, miRISC half-lives vary across individual miRNAs in a cell-type-dependent manner, suggesting that cell-specific factors modulate miRISC turnover rates (Kingston and Bartel, 2019). As discussed in the following section, miRNA loading efficiency into RISC is also regulated by specific epitranscriptomic modifications.

## 6 Epitranscriptome in the regulation of miRNA processing, stability, and functional efficacy

Epitranscriptomic modifications of nucleosides, such as N6-methyladenosine (m6A), adenosine-to-inosine editing (A-to-I), 5-methylcytosine (m5C), 7-methylguanosine (m7G), and others, represent an additional layer of post-transcriptional regulation of miRNA expression. In this review, we focus on examples of epitranscriptomic modifications that result in differential expression of miRNAs encoded within a single polycistronic transcript (Table 2).

**TABLE 2:** Regulation of clustered miRNA maturation by epitranscriptomic modifications.

miRNA/precursor	Enzyme	Effect of the modification	Experimental model/cell or tissue type	References
A-to-I editing				
pri-miR-142	ADAR1/ADAR2	Inhibition of processing by Drosha and degradation by Tudor-SN.	*In vitro*, HEK293, spleens and thymuses of mice	(Yang et al., 2006)
pri-miR-1-1	ADAR1/ADAR2	ADARs modified the pri-miRNA, suggesting subsequent inhibition of its processing by Drosha	*In vitro*	(Yang et al., 2006)
pri-miR-143	ADAR1/ADAR2	ADARs modified the pri-miRNA, suggesting subsequent inhibition of its processing by Drosha	*In vitro*	(Yang et al., 2006)
miR-136	ADAR1	ADAR1 knockdown enhances miR-136 maturation, indicating that ADAR1-dependent editing negatively regulates precursor processing. The precise processing step is not specified	RAW264.7 macrophages	(Li et al., 2018a)
let-7d	ADAR1	ADAR1 knockdown enhances let-7d maturation, indicating that ADAR1-dependent editing negatively regulates precursor processing. The precise processing step is not specified	RAW264.7 macrophages	(Li et al., 2018a)
miR-221	ADAR2	ADAR2 suppresses miR-221 maturation. The precise processing step is not specified	Glioblastoma cell models and mouse brain *in vivo*	(Tomaselli et al., 2015)
miR-222	ADAR2	ADAR2 suppresses miR-222 maturation. The precise processing step is not specified	Glioblastoma cell models and mouse brain *in vivo*	(Tomaselli et al., 2015)
miR-21	ADAR2	ADAR2 suppresses miR-21 maturation. The precise processing step is not specified	Glioblastoma cell models and mouse brain *in vivo*	Tomaselli et al., 2015; PMID: 25582055
pri-miR-100	ADAR	Editing at position −6 promotes Drosha-mediated processing, whereas editing at +41 and/or +42 inhibits it. When both types of editing coexist, the stimulatory −6 effect predominates, increasing miR-100 abundance	*Drosophila* BG3c2 and Kc-167 cells, *in vitro*	(Chawla and Sokol, 2014)
pri-miR-125	ADAR	Editing at position +43 destabilizes the entire pri-let-7-C polycistronic transcript, coordinately reducing miR-100, let-7, and miR-125 levels	*Drosophila*BG3c2 and Kc-167 cells, *in vitro*	(Chawla and Sokol, 2014)
pri-miR-197	not specified	The editing at the +14 site stimulates Drosha-mediated processing	*In vitro*	(Kawahara et al., 2008)
pri-let-7g	not specified	Editing at the +14 site did not affect the Drosha cleavage step, but suppressed the Dicer cleavage step.	*In vitro*	(Kawahara et al., 2008)
pri-miR-497	ADAR; specific paralogue not specified	Stimulates Drosha-mediated processing	Mouse brain, HEK293	(Vesely et al., 2014)
miR-497	ADAR; specific paralogue not specified	Editing at nucleotide 2 within the seed region may fine-tune the pro-apoptotic activity of miR-497 in neurons by altering its target-recognition specificity	Mouse brain; neurons	(Vesely et al., 2014)
pre-miR-151	ADAR1	Dicer–TRBP-mediated processing of pre-miRNA	*In vitro*, mouse embryos, human amygdala, mouse cerebral cortex and mouse lung	(Kawahara et al., 2007a)
miR-27a	ADAR1 and ADAR2	Non-canonical ADAR-dependent regulation, independent of A-to-I editing ADAR1 and ADAR2 physically associate with Dicer and promote the production of mature miR-27a	Huh-7	(Qi et al., 2017)
miR-376a-5p	ADAR2	A-to-I editing at position +4 of the seed region alters miR-376a-5p target-recognition specificity	Cerebral mouse cortex	(Kawahara et al., 2007b)
miR-376a-3p	ADAR; specific paralogue not specified	A-to-I editing at position +6 of the seed region A 6% decrease in editing correlates with marked remodeling of mRNA target networks. Inhibition of the edited isoform induces mitochondrial membrane hyperpolarization	Hippocampus of patients with temporal lobe epilepsy; iPSC-derived neurons	(How Lau et al., 2023)
miR-200b	ADAR1	Attenuates the capacity of miR-200b to repress ZEB1 expression	TPC1 and Cal62 thyroid cancer cells	(Ramírez-Moya et al., 2020)
m6A				
miR-99b		METTL3 depletion reduces level of the mature miRNA; decreases the abundance of the miRNAs in extracellular vesicles	DKO-1 human colorectal cancer cells	(Abner et al., 2021)
let-7e	METTL3	METTL3 depletion reduces level of the mature miRNA.	DKO-1 human colorectal cancer cells, human endothelial cells	(Abner et al., 2021; Chamorro-Jorganes et al., 2021)
miR-125a	METTL3	METTL3 depletion reduces level of the mature miRNA.	DKO-1 human colorectal cancer cells	(Abner et al., 2021)
miR-100	METTL3	METTL3 depletion reduces level of the mature miRNA; decreases the abundance of the miRNAs in extracellular vesicles	DKO-1 human colorectal cancer cells	(Abner et al., 2021)
miR-125b	METTL3	METTL3 depletion reduces level of the mature miRNA.	DKO-1 human colorectal cancer cells	(Abner et al., 2021)
Members of the miR-17–92 cluster	METTL3	METTL3 depletion reduces levels of all members of this cluster	Human endothelial cells	(Chamorro-Jorganes et al., 2021)
pri-miR-93	METTL3	Accelerates processing by Microprocessor and increases mature miR-93-5p abundance	Human prostate cancer cells; HepG2 and SMMC-7721 liver cancer cells	(Qi et al., 2023; Sun et al., 2023; Wang et al., 2025b)
pri-miR-93	METTL14	Stimulates pri-miRNA processing	Non-small cell lung cancer tissues and cells	(Qian et al., 2024)
pri-miR-25	METTL3	Accelerates processing by Microprocessor and increases mature miR-25-3p abundance	Human prostate cancer cells	(Qi et al., 2023)
pri-miR-125b-2/pre-miR-125b-2	NSun2	Inhibits both Drosha- and Dicer-mediated processin	IDH4 cells	(Yuan et al., 2014)
miR-125b	NSun2	Attenuates recruitment of miR-125b to the RISC.	IDH4 cells	(Yuan et al., 2014)
miR-200c	Not specified	Alters the ability to bind Ago proteins and suppress target genes	Gastrointestinal cancer	(Konno et al., 2019)
miR-17-5p	Not specified	Molecular modeling indicates that m6A produces substantial conformational changes in RISC and reduces target recognition efficiency	Molecular modeling	(Yuan et al., 2014)
let-7a-5p	Not specified	Molecular modeling indicates that m6A produces substantial conformational changes in RISC and reduces target recognition efficiency	Molecular modeling; no cell type specified	(Yuan et al., 2014)
m5C				
miR-16-5p	Dnmt3a–Ago4 complex	Impedes formation of the miRNA–mRNA duplex and weakens repression of target genes	Human glioblastoma cells	(Cheray et al., 2020)
miR-181a-5p	Dnmt3a–Ago4 complex	Impedes formation of the miRNA–mRNA duplex and weakens repression of target genes	Human glioblastoma cells	(Cheray et al., 2020)
m7G				
pri-let-7e/pre-let-7e	METTL1	Enhances Drosha-mediated processing	A549 cells	(Pandey et al., 2015; Pandolfini et al., 2019)
pre-miR-92b	METTL1	Enhances Dicer-mediated processing	A549 cells, HEK293	(Mirihana Arachchilage et al., 2015; Pandolfini et al., 2019)
miR-125a	METTL1	Putative m7G-dependent regulation METTL1 suppression reduces miR-125a expression	A549 cells	(Pandolfini et al., 2019)
let-7a	METTL1	Putative m7G-dependent regulation METTL1 suppression reduces let-7a expression	A549 cells	(Pandolfini et al., 2019)
let-7b	METTL1	Putative m7G-dependent regulation METTL1 suppression reduces let-7b expression	A549 cells	(Pandolfini et al., 2019)
let-7c	METTL1	Putative m7G-dependent regulation METTL1 suppression reduces let-7c expression	A549 cells	(Pandolfini et al., 2019)
miR-23b	METTL1	Putative m7G-dependent regulation METTL1 suppression reduces miR-23b expression	A549 cells	(Pandolfini et al., 2019)
5′-phospho-dimethylation, 5′-Pme2				
pre-miR-145	BCDIN3D	Prevents pre-miRNA recognition by Dicer	MDA-MB-231 and MCF-7 cells	(Xhemalce et al., 2012)
pre-miR-23b	BCDIN3D	Prevents pre-miRNA recognition by Dicer	MDA-MB-231 and MCF-7 cells	(Xhemalce et al., 2012)
3′-terminal 2′-O-methylation				
miR-143-3p		Putative 3′-terminal 2′Ome -dependent regulation of degradation	Plasma samples of patients with pancreatic ductal adenocarcinoma	(Yang et al., 2024)
miR-92a-3p		Putative 3′-terminal 2′Ome -dependent regulation of degradation	Plasma samples of patients with pancreatic ductal adenocarcinoma	(Yang et al., 2024)
miR-99b-5p		Putative 3′-terminal 2′Ome -dependent regulation of degradation	Plasma samples of patients with pancreatic ductal adenocarcinoma	(Yang et al., 2024)

### 6.1 Adenosine-to-inosine editing (A-to-I)

The role of A-to-I editing by adenosine deaminases ADAR1 and ADAR2 in miRNA biogenesis was first demonstrated by Yang et al. (2006). ADAR-mediated editing of pri-miRNAs disrupts their secondary structure through the formation of wobble I-U pairs, thereby inhibiting Microprocessor-mediated cleavage. Unprocessed precursors are subsequently degraded by the endonuclease Tudor-SN (TSN), which recognizes inosine-containing double-stranded RNA. Among the eight pri-miRNAs tested *in vitro*, ADARs selectively modified pri-miR-142, pri-miR-223, pri-miR-1-1, and pri-miR-143 (Yang et al., 2006). Notably, miR-142, miR-1-1, and miR-143 belong to respective clusters, suggesting that ADAR-mediated editing could contribute to differential regulation within polycistronic transcripts. For pri-miR-142, A-to-I editing was shown to inhibit Drosha-mediated processing in HEK293 cells. Edited pri-miR-142 can subsequently be degraded by Tudor-SN (Yang et al., 2006). There is also evidence that ADAR1 and ADAR2 appear to target partially distinct subsets of miRNA precursors in mice (Kawahara et al., 2008) and compete for binding to shared substrates (Vesely et al., 2014).

Li J. et al. (2018) showed that ADAR1 knockdown enhances the maturation of the clustered miRNAs miR-136 and let-7d, suggesting that A-to-I editing negatively regulates the processing of their precursors. Both in glioblastoma cell models and in the mouse brain *in vivo*, ADAR2 suppresses the maturation of oncogenic miRNAs, including miR-221, miR-222, and miR-21, whose precursors contain multiple A-to-I editing sites (Tomaselli et al., 2015). Given that ADAR2 expression and activity are commonly compromised in glioblastoma, its restoration attenuates cellular proliferation and invasiveness by de-repressing the tumor suppressor proteins p27^Kip1^ and PDCD4, direct targets of miR-221/222 and miR-21, respectively (Tomaselli et al., 2015).

In *Drosophila*, the evolutionarily conserved let-7-C cluster comprises miR-100, let-7, and miR-125 which are co-transcribed as a single transcript yet, as noted previously, accumulate at markedly divergent steady-state levels *in vivo*. Chawla and Sokol (2014) revealed that A-to-I editing of the pri-let-7-C represents another layer of regulation underlying this uncoupled expression. Specifically, editing of pri-miR-100 at position −6 promotes Drosha-mediated processing, whereas editing at positions +41 and/or +42 reduces it. When both editing events occur, the stimulatory effect at position −6 predominates, resulting in increased miR-100 levels. Editing of pri-miR-125 at position +43 destabilizes the entire primary transcript, reducing levels of all three miRNAs in the cluster. This may serve as a mechanism for rapid removal of the entire miRNA set from the system (Chawla and Sokol, 2014). Taken together, these findings demonstrate that A-to-I editing can exert opposing, and even mutually exclusive, regulatory outcomes dictated not by substrate identity, but by the precise position of the modification. Furthermore, the destabilizing effect of pri-miR-125 editing at position +43 represents a qualitatively distinct layer of control: rather than driving the uncoupled processing of an individual cluster member, it triggers the coordinated degradation of the entire polycistronic transcript. This exemplifies how epitranscriptomic modifications can operate both locally (by fine-tuning the biogenesis of a specific hairpin) and globally, by dictating the fate of the intact primary transcript. The latter mechanism temporarily abrogates differential expression altogether, as all co-transcribed miRNAs are lost simultaneously. The dynamic balance between these two editing-mediated modes likely underpins the progressive shifts in let-7-C miRNA expression profiles during *Drosophila* development in parallel with rising ADAR levels.

A stimulatory effect of A-to-I editing on Drosha-mediated processing has also been documented for cluster-derived pri-miR-197 (Kawahara et al., 2008) and pri-miR-497 (Vesely et al., 2014).

In addition to affecting Drosha-mediated processing of pri-miRNAs, A-to-I editing can also influence Dicer-mediated cleavage of pre-miRNAs. For example, editing of the miR-151 precursor does not disrupt Drosha–DGCR8 cleavage but completely blocks subsequent processing by the cytoplasmic Dicer–TRBP complex (Kawahara et al., 2007a). Similarly, editing of pri-let-7g at the +14 site does not affect the Drosha cleavage step but suppresses Dicer-mediated processing (Kawahara et al., 2008).

ADAR1 and ADAR2 also regulate miRNA expression through a non-canonical, A-to-I editing-independent mechanism. For example, they can physically associate with Dicer and promote the production of mature clustered miR-27a without increasing precursor levels (Qi et al., 2017).

Furthermore, A-to-I editing within pri-miRNAs at positions corresponding to the seed region of mature miRNAs can alter their target repertoire, since cellular machinery reads inosine as guanosine. This type of target reprogramming, resulting from a single nucleotide substitution in the seed region, was first discovered for miR-376a-5p, a member of the mega-cluster C14MC (Kawahara et al., 2007b). Specifically, editing at position +4 redirects miR-376a-5p to selectively silence *PRPS1*, encoding a key enzyme in the purine catabolic pathway leading to uric acid synthesis. In Adar2-knockout mice, the loss of this edit results in a two-fold upregulation of PRPS1 and a corresponding twofold accumulation of uric acid in the cerebral cortex.

A-to-I editing also attenuates the ability of the clustered miR-200b to suppress *ZEB1* expression, though the specific site of modification has not been detailed (Ramírez-Moya et al., 2020). In addition, A-to-I editing can influence strand selection for RISC loading. Editing of one pre-miRNA arm promotes preferential loading of the miRNA from the opposite arm (Li L. et al., 2018).

A quantitative atlas of A-to-I editing sites, generated by profiling hundreds of human and animal pri-miRNA samples, revealed that approximately 80% of all transcribed pri-miRNAs undergo editing in at least one tissue type, predominantly in neuronal tissues (Li L. et al., 2018). Although this phenomenon is widespread, the editing frequency at any given site is generally low (<5%) (Li L. et al., 2018). However, a formally low average editing frequency at a specific site does not preclude its functional significance. First, metrics derived from bulk tissue profiling average signals across heterogeneous cell populations; within distinct cellular subpopulations (such as specific neuronal subtypes or cells under defined stress conditions), the actual editing frequency at a given site may be substantially higher than the bulk average. Second, for modifications situated within mature miRNA seed regions, the biologically critical event may be the target repertoire switch itself, rather than the average editing frequency across the entire population. Third, different tissues exhibit varying degrees of “dependence” on A-to-I editing. As noted above, this modification is most prominent in brain tissue. In adult mice, editing levels in the brain are considerably higher than in embryos (as measured by the number of edited miRNAs) (Ekdahl et al., 2012; Vesely et al., 2014) highlighting a pivotal role for A-to-I editing in brain development. Notably, adult animals display a marked enrichment of A-to-I editing sites specifically within miRNA seed regions, accounting for at least 64% of all detected editing events (Vesely et al., 2014; Paul et al., 2017). Fourth, editing frequencies for select pri-miRNAs and mature miRNAs can be exceptionally high at precisely defined positions. For example, mature miR-497 is edited at position +2 of its seed region with an efficiency of 52%. Given that miR-497 participates in regulating neuronal cell death in the murine brain, this editing event may finely modulate its pro-apoptotic activity in neurons (Vesely et al., 2014). Similarly, pri-miR-376a2, pri-miR-376b, and pri-miR-376c (formerly designated pri-miR-368), all encoded within the same cluster, exhibit near-complete (∼100%) editing at position +44 in the human cortex and medulla, a phenomenon absent in non-neural tissues (Kawahara et al., 2007b). This site corresponds to position +6 of the seed region in miRNAs derived from the 3′arm of the precursor hairpin. Such near-stoichiometric frequency underscores the functional importance of editing at this site in the brain. Indeed, edited miR-376b-3p was recently shown to repress the enzyme 4-aminobutyrate aminotransferase (ABAT), which catabolizes the neurotransmitter gamma-aminobutyric acid (GABA), thereby modulating GABAergic neurotransmission in the brain (Widmark et al., 2022). Fifth, even modest shifts in editing frequency can dramatically alter downstream mRNA target landscapes. For instance, a mere 6% reduction in A-to-I editing at position +6 within the seed region of miR-376a-3p correlates with substantial remodeling of target mRNA networks in the hippocampus of patients with temporal lobe epilepsy (How Lau et al., 2023). Furthermore, inhibiting the edited isoform of miR-376a-3p in iPSC-derived neurons induces mitochondrial membrane hyperpolarization (How Lau et al., 2023), which may represent a metabolic reprogramming mechanism conferring neuronal resilience against metabolic and oxidative stress in epilepsy, or otherwise reflect an endogenous compensatory response.

### 6.2 N6-methyladenosine (m6A)

m6A methylation is among the most extensively studied epitranscriptomic modifications and influences virtually every stage of RNA metabolism, including pre-mRNA splicing, nuclear export, translation, and miRNA biogenesis (Khan et al., 2024c). In miRNA pathways, as in other RNA regulatory contexts, m^6^A functions as an important post-transcriptional regulatory mark (Khan et al., 2024c). As in mRNAs, the deposition of m6A marks on pri-miRNA transcripts is carried out by the methyltransferase METTL3. These modifications serve as recognition signals for DGCR8, promoting recruitment of the endonuclease Drosha (Alarcón et al., 2015b). Abner et al. (2021) showed that METTL3 depletion in DKO-1 human colorectal cancer cells reduces levels of a specific subset of miRNAs containing consensus m6A sites, including all members of the miR-99b∼let-7e∼miR-125a cluster (to varying degrees) and miR-100 and miR-125b from the miR-100∼let-7a∼miR-125b cluster. This indicates that m6A methylation selectively enhances maturation of miRNAs whose precursor sequences contain methylation sites (RRACH motifs, where R = G/A, H = A/C/U). Notably, loss of m6A also decreased the abundance of these miRNAs, as well as some others, in extracellular vesicles, suggesting that m6A methylation is important for miRNA secretion (Abner et al., 2021). Positive effects of m6A on maturation were also observed for let-7e of the miR-99b-125a cluster and for members of the miR-17–92 cluster in human endothelial cells (Chamorro-Jorganes et al., 2021). Furthermore, METTL3-mediated m6A methylation has been shown to accelerate the maturation of clustered pri-miR-93 (Qi et al., 2023; Wang Y. et al., 2025) and pri-miR-25 (Qi et al., 2023), thereby increasing the expression of mature miR-93-5p and miR-25-3p, respectively. In addition, m^6^A methylation facilitates more uniform pri-miRNA cleavage by Drosha, thereby decreasing the probability of miRNA isoform generation (Alarcón et al., 2015b).

The protein HNRNPA2B1 (hnRNP A2/B1) serves as a nuclear adapter that recognizes m6A marks on both mRNAs and pri-miRNAs (Alarcón et al., 2015a). By binding to m6A-containing pri-miRNAs, HNRNPA2B1 recruits the DGCR8/DROSHA complex, thereby promoting efficient processing (Alarcón et al., 2015a). Specifically, HNRNPA2B1, acting as a nuclear m6A reader, together with METTL3 and DGCR8, has been shown to enhance the maturation of pri-miR-93 into miR-93-5p in prostate cancer cells (Qi et al., 2023; Sun et al., 2023). Moreover, one study emphasized the importance of HNRNPA2B1 phosphorylation at Thr20 for this process, as this modification stabilizes the protein (Qi et al., 2023).

The methyltransferase METTL14 has also been reported to mediate m6A modification of pri-miR-93, which in turn stimulates its maturation and is accompanied by increased expression of mature miR-93-5p in tissues and cells of non-small cell lung cancer (Qian et al., 2024).

Yuan and colleagues (Yuan et al., 2014) revealed that the RNA methyltransferase NSun2, traditionally characterized as a cytosine-5 writer, also catalyzes m6A deposition on pri-miR-125b-2 within the polycistronic miR-100∼let-7a∼miR-125b cluster. This methylation impairs both Drosha- and Dicer-mediated cleavage steps during its biogenesis. Interestingly, this inhibitory effect was stronger for pri-/pre-miR-125b-2 than for pri-/pre-miR-125b-1. Notably, these miR-125b precursors belong to different clusters. These findings indicate the existence of context-dependent regulation, potentially influenced by both the genomic location of the miRNA precursor and cell type. Importantly, this stands in sharp contrast to the stimulatory effect of METTL3-mediated m6A modification on pri-miR-125b maturation noted earlier (Abner et al., 2021). Although the consensus motifs targeted by METTL3 (RRACH) and NSun2 (RRACH and AAC) intersect, pri-miR-125b-2 contains considerably more putative NSun2 methylation sites (6 vs. 2). Consequently, the conflicting biological outcomes might stem from differences in site-specific positioning or overall modification stoichiometry, features that could be dynamically fine-tuned in a cell-context-dependent manner. However, the exact molecular determinants that dictate the opposing downstream fates imparted by METTL3-versus NSun2-mediated methylation remain to be elucidated. The contribution of methylation at individual sites, the cumulative effect of multiple methylation events, and the role of the relevant writer enzyme can be assessed using several complementary approaches: (1) site-specific m^6^A editing using dCas13-fusion constructs containing METTL3 or NSun2, which allows to install the modification at a defined nucleotide within pri-miR-125b-2 (Wilson et al., 2020); (2) site-directed mutagenesis of individual adenosines within the RRACH and AAC motifs of pri-miR-125b-2 (A→G substitution prevents methylation at these sites), followed by transfection of mutant constructs into cells overexpressing either METTL3 or NSun2; and (3) assessment of cleavage efficiency by Drosha *in vitro* using a purified Microprocessor complex and a synthetic pri-miR-125b-2 transcripts carrying defined methylation patterns.

m6A modification of mature miRNAs can also alter their ability to bind to Ago protein and suppress target genes, as shown for the clustered miRNAs miR-200c (Konno et al., 2019). Also NSUN2-mediated methylation attenuates recruitment of miR-125b to the RISC, thereby impairing miR-125b-mediated gene silencing (Yuan et al., 2014). Molecular modeling for miR-17-5p and let-7a-5p demonstrated that m6A induces large conformational changes in the RISC complex, reducing target recognition efficiency (Yuan et al., 2014). Notably, miRNA methylation is enhanced upon epidermal growth factor (EGF) stimulation, suggesting a regulated system for dynamic miRNA modification.

### 6.3 5-Methylcytosine (m5C)

Methylation of cytosine at the 5th position (m5C) can also influence miRNA target binding. (Cheray et al., 2020) demonstrated that several mature miRNAs, including the clustered miR-16-5p and miR-181a-5p in human glioblastoma cells, contain m5C. This modification is established by a complex of the methyltransferase Dnmt3a and Ago4. The presence of m5C physically impedes formation of a duplex between the miRNA and the 3′UTR of the mRNA target, thereby reducing the repressive functions of the miRNA.

### 6.4 7-Methylguanosine (m7G)

Some miRNA precursor hairpins can form alternative structures, such as G-quadruplexes, which act as structural barriers that impede Drosha- and Dicer-mediated cleavage, lowering levels of functional mature miRNAs, including clustered let-7e and miR-92b (Mirihana Arachchilage et al., 2015; Pandey et al., 2015; Pandolfini et al., 2019). Bioinformatic analyses suggest that approximately 13%–23% of all human pre-miRNAs have high GC content and potential quadruplex motifs (Pandey et al., 2015). For pri-let-7e, m7G modification by the methyltransferase METTL1 prevents G-quadruplex formation, enhancing Drosha-mediated cleavage efficiency (Pandolfini et al., 2019). Suppression of METTL1 reduces expression of several other miRNAs, including clustered miRNAs miR-125a, let-7a, let-7b, let-7c, and miR-23b (Pandolfini et al., 2019), suggesting that this mechanism regulates maturation of these miRNAs.

### 6.5 5′phospho-dimethylation (5′-Pme2)

During miRNA processing, Drosha generates a 5′-monophosphate end, whose negative charge is critical for recognition by Dicer and Ago2, ensuring processing stability and accuracy (Gebert and MacRae, 2018). The RNA methyltransferase BCDIN3D dimethylates the 5′-monophosphate of certain pre-miRNAs (Xhemalce et al., 2012). Methylation of both oxygen atoms neutralizes the phosphate’s negative charge, preventing pre-miRNA recognition by Dicer and ultimately reducing mature miRNA production. This mechanism was first described for clustered pre-miR-145 but also occurs for clustered pre-miR-23b and other miRNAs precursors (Xhemalce et al., 2012).

### 6.6 2′-O-methylation of the 3′-terminal ribose residue (3′-terminal 2′Ome)

Epitranscriptomic modifications can also regulate mature miRNA levels by influencing their stability. In plants and *Drosophila*, 2′-O-methylation of the 3′-terminal ribose (3′-terminal 2′Ome), catalyzed by HEN1 and homologs, protects miRNAs from 3′tailing (primarily uridylation and subsequent degradation) and 3′→5′exonucleolytic degradation (Ji and Chen, 2012). Interestingly, target transcripts can modulate the stability of guiding miRNAs, either stabilizing them or promoting their decay. In mammals, miRNAs generally lack 3′-terminal 2′-O-methylation under normal conditions; however, this modification may occur in pathological settings, as reported for miR-21-5p in non-small cell lung cancer, where it enhances miRNA stability and Ago2 association (Liang et al., 2020). In mammals, the methyltransferase HENMT1 installs this mark. Whether 3′-terminal 2′-O-methylation occurs in clustered animal miRNAs has not yet been systematically examined. However, circulating 2′-O-methylated miR-143-3p, miR-92a-3p, and miR-99b-5p have been detected in patients with pancreatic ductal adenocarcinoma (Yang et al., 2024).

In summary, the epitranscriptome orchestrates the differential expression of clustered miRNAs at all biogenesis milestones—from Drosha-mediated primary cleavage (A-to-I, m6A, m7G) through Dicer processing (5′-Pme2; A-to-I and m6A in some cases) to mature miRNA turnover (3′-terminal 2′Ome), RISC-dependent targeting (A-to-I, m5C, m6A), and and extracellular vesicle sorting/secretion (m6A). Whereas the genetic and post-transcriptional regulators detailed earlier (alternative promoter usage, splicing, cluster assistance, and RBPs) largely impose binary, all-or-nothing switches on hairpin availability and cleavage, epitranscriptomic marks confer rheostatic, graded, and predominantly reversible control (barring A-to-I editing). Consequently, epitranscriptomic mechanisms serve as an agile molecular platform for the acute, signal-dependent rewiring of clustered and non-clustered miRNA repertoires.

Dysregulated epitranscriptomic modifications in miRNAs and pri-miRNAs are prevalent across multiple cancers and other disorders, suggesting central roles in disease mechanisms. However, the modifications discussed so far have almost exclusively been studied independently on seemingly separate precursor pools. The potential for combinatorial regulation or competitive deposition of different marks on the exact same pri-miRNA molecule is therefore largely uncharacterized. A lack of systematic studies profiling multi-mark epitranscriptomic combinations on single pri-miRNA transcripts constitutes a notable gap and a promising direction for upcoming work.

## 7 Degradation of precursors and mature miRNAs as a source of differential stability of clustered miRNAs

In addition to covalent epitranscriptomic marks and the other regulatory mechanisms discussed above, targeted decay mediated by dedicated ribonucleases represents a critical checkpoint governing mature miRNA abundance. Because the decay kinetics of individual miRNAs can diverge by over tenfold (Kim et al., 2025), programmed degradation constitutes an independent regulatory mechanism driving intra-cluster expression divergence that cannot be accounted for solely by differential Drosha/Dicer cleavage efficiencies.

The most extensively characterized pathway for the targeted degradation of miRNA precursors involves the RNA-binding proteins Lin28A and Lin28B, which selectively engage specific miRNA precursors, including clustered pre-miR-143, pre-miR-200c, and members of the pre-let-7 family (where 11 out of 12 pre-miRNAs are clustered (Lee et al., 2016; Ware et al., 2022)). Upon binding, Lin28 proteins recruit the terminal uridylyltransferases TUT4, and potentially TUT7, to promote the uridylation of the precursors’ 3′ends (Heo et al., 2008; 2009). This uridylation prevents Dicer-mediated processing and targets the precursors for degradation (Heo et al., 2008). Additionally, Lin28 proteins are thought to compete directly with Dicer for pre-let-7 binding (Rybak et al., 2008). In vertebrates, Lin28 binding specificity is primarily driven by a GGAG motif within the terminal loops of the pre-miRNAs (Heo et al., 2009). However, in human cells, variations of this motif (such as GAAG, GGUG/GGCG, and UGXG) can also occur in certain pre-miRNAs, including clustered pre-let-7b, pre-let-7a-3, and pre-miR-98 (Heo et al., 2009). Furthermore, Lin28A activity is subject to post-translational regulation. Sumoylation of Lin28A at lysine 15 (K15) significantly enhances its binding affinity for pre-let-7. This modification effectively blocks pre-let-7 processing, thereby suppressing the production of mature let-7 miRNAs (Dou et al., 2020). Thus, through sequence-specific interactions, RBPs like Lin28 can selectively regulate the degradation of individual miRNA precursors.

For several other miRNAs, turnover is governed by an adenylation-dependent decay route rather than terminal uridylation. Non-canonical polyadenylation by PAPD5 (TUT3/GLD4/TENT4B/TRF4-2) tags specific pre-miRNAs and mature strands with short oligo(A) tails, priming them for rapid clearance by 3′→5′exoribonucleases DIS3L/DIS3L2 (Shukla et al., 2019). By trimming these oligo(A) extensions, the deadenylase PARN acts as a protective stabilizer for distinct miRNA precursors and mature products (Shukla et al., 2019; Kyritsis et al., 2026). However, supraphysiological PARN levels can also promote 3′degradation (e.g., of miR-125a-5p), though less efficiently than DIS3L (Tomasello et al., 2024). The kinetic balance between PAPD5 tailing and PARN trimming thereby tunes individual miRNA half-lives. Auxiliary RNA-binding proteins, such as CUGBP1, can further confer transcript specificity to PARN-mediated regulation, as reported for miR-122 (Katoh et al., 2015). Beyond bulk turnover, this cyclical addition and removal of non-templated 3′adenines by PAPD5 and PARN generates and stabilizes specific 3′isomiRs, including clustered miR-125a-5p (Tomasello et al., 2024). Importantly, non-seed sequence and length variations at the 3′end of miR-125a-5p can profoundly reshape target engagement and repressive efficacy (Tomasello et al., 2024).

A diverse cohort of ribonucleases mediates miRNA clearance, encompassing the 5′→3′-exoribonucleases XRN1 and XRN2, the 3′→5′-exoribonucleases PNPT1 and ERI1, as well as the endoribonuclease Tudor-SN/SND1 (Kim et al., 2025). Nevertheless, their distinct contributions and potential crosstalk remain poorly understood. Because miRNA stability varies across distinct cellular contexts, lineage-specific factors or regulatory mechanisms likely govern individual decay pathways in a cell-type-dependent manner. Intriguingly, beyond its established role in degrading mature miRNAs (such as members of the let-7 family (Chatterjee and Großhans, 2009; Chatterjee et al., 2011)), the 5′→3′-exonuclease XRN2 can directly bind pre-miR-10a and accelerate its maturation via a Dicer-independent pathway, thereby promoting the epithelial–mesenchymal transition (EMT) and metastasis in non-small cell lung cancer (Zhang et al., 2017).

Taken together, the diverse decay pathways targeting precursor and mature miRNAs support the addition of a dedicated tier to our regulatory taxonomy: selective clearance, acting concurrently with rather than sequentially after Microprocessor and Dicer cleavage. Whereas regulatory RBPs and epitranscriptomic marks primarily govern processing competence, exonucleolytic degradation sets the steady-state half-life of processed and unprocessed RNA species. Because cleavage efficacy and degradation rates can diverge autonomously between adjacent hairpins in a shared polycistron, intra-cluster mature miRNA stoichiometry should be viewed through the lens of kinetic competition between these two orthogonal processes, rather than through biogenesis efficiency in isolation.

## 8 Conclusions and final remarks

Polycistronic pri-miRNAs can yield mature miRNAs belonging to the same or distinct miRNA families. These clustered miRNAs may share common targets and synergistic functions or, conversely, regulate target genes with opposing roles. Therefore, the balance between the coordinated and differential expression of individual miRNAs within a cluster can dictate completely different biological outcomes. Thus, the differential expression of miRNAs co-encoded within a single cluster does not represent processing infidelity or biological noise; rather, it constitutes a tightly regulated evolutionary mechanism that enables the cell to extract multiplexed regulatory outputs from a single genomic locus.

As summarized across this review, cells exploit a multifaceted repertoire of mechanisms to uncouple individual miRNAs from a shared polycistronic transcript, allowing intra-cluster fates to diverge at every step of the biogenesis pathway (Figure 6). Structuring these decoupling mechanisms by their response kinetics and reversibility provides a valuable conceptual matrix. At one extreme, alternative transcription and splicing are genetically hardwired, acting as binary switches governed by transcriptional and splicing machinery; they represent slow-responding, poorly reversible regimes over the course of a cell cycle. At the other extreme, dynamic epitranscriptomic marks and RBP activities modulated by post-translational modifications (e.g., KSRP SUMOylation, HNRNPA2B1 phosphorylation) adjust rapidly (minutes to hours) to acute signaling cues in a predominantly reversible manner without genetic alterations. Cluster assistance straddles these two paradigms: while local secondary structures impose static constraints, protein-assisted mechanisms (driven by ERH and SAFB) are susceptible to fluctuations in factor stoichiometry. We posit that highly reversible regulatory tiers orchestrate rapid, acute stress-induced reconfiguration of miRNA cluster profiles (e.g., during hypoxia or oxidative stress), contrasting with the inertia of genome-hardwired layers. Validating this framework through comparative time-resolved profiling of intra-cluster mature miRNA ratios across acute exposure versus prolonged adaptation represents a key priority for future work.

**FIGURE 6:**
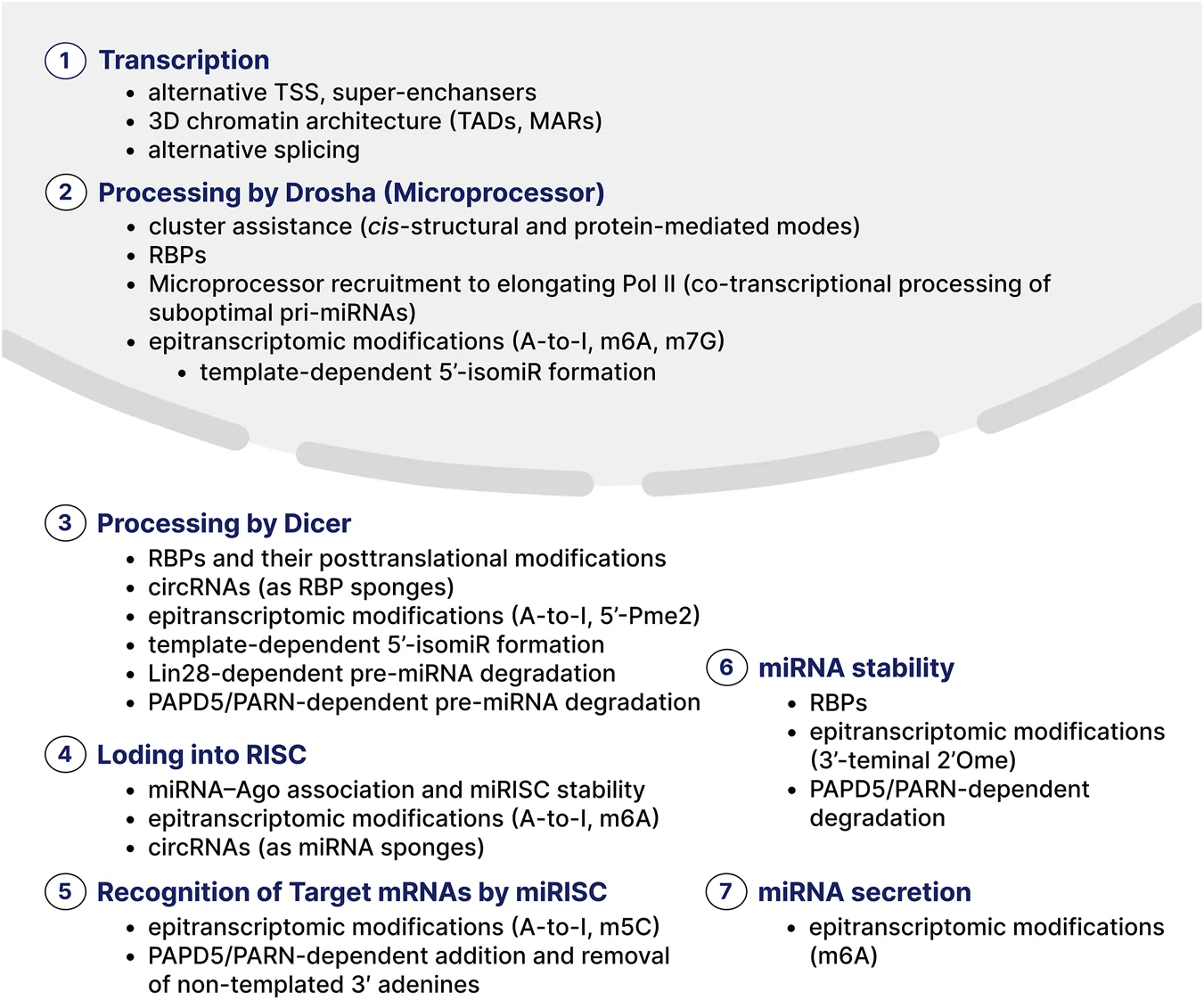
Decoupling mechanisms governing the differential expression of polycistronic miRNAs across the biogenesis pathway. Schematic overview of the regulatory layers that uncouple the expression of individual miRNAs encoded within a single primary transcript. These mechanisms operate across multiple biographical stages, encompassing alternative transcription start site (TSS) selection, differential Microprocessor cleavage, RNA-binding protein (RBP) modulation, epitranscriptomic modifications, Dicer processing fidelity, and selective mature miRNA decay.

Selective modulation of individual miRNAs within polycistronic clusters could represent a promising therapeutic strategy for diverse diseases. Targeted interventions (including small molecules or antisense oligonucleotides directed against RBP-binding sites, specific splice sites, or writer or reader proteins that install or recognize epitranscriptomic marks) could, in principle, enable artificial reprogramming of the expression profiles of disease-relevant miRNA clusters. For instance, miR-106b and miR-93 (encoded by the miR-106b-25 cluster) curb pancreatic cancer immunosuppression through PD-L1 downregulation. Several decoupling mechanisms governing this polycistron are already defined: an internal alternative promoter generates a pri-miR-25-deficient isoform (Ramalingam et al., 2014); alternative splicing reduces pri-miR-25 incorporation via exon extension (Agranat-Tamir et al., 2014); METTL3-dependent m6A deposition stimulates miR-93 biogenesis (Qi et al., 2023); and HNRNPA2B1 phosphorylation enhances its stability and efficacy as m6A reader promoting pri-miR-93 maturation (Qi et al., 2023). Based on these findings, a therapeutic shift in the balance toward miR-106b/miR-93 might, in principle, be achieved through one or a combination of the following approaches: modulation of splicing factors that control inclusion or exclusion of the exon containing pri-miR-25; selective stabilization of the transcript initiated from the proximal promoter; activation of CSNK1D kinase, which phosphorylates HNRNPA2B1, using small-molecule compounds; or selective inhibition of phosphatases that dephosphorylate HNRNPA2B1. However, further investigation is required to determine which of these approaches, or which combination thereof, would be most effective, as none has yet been experimentally validated as a therapeutic strategy. Moreover, the feasibility of selectively altering miRNA stoichiometry within a cluster *in vivo* without causing substantial systemic perturbations remains to be established.

Conversely, the biology of miR-132 in Alzheimer’s disease (AD) highlights a striking blind spot in our mechanistic understanding of cluster decoupling. Arising from the conserved miR-132∼miR-212 locus, miR-132 undergoes profound downregulation across intermediate-to-late Braak stages of AD and other neurodegenerative disorders (Salta and De Strooper, 2017). Depletion of miR-132 accelerates AD progression through multifaceted mechanisms: it facilitates amyloid-beta (Aβ) accumulation, TUA protein pathology, promotes neuronal loss, and compromises neurogenesis, synaptic transmission, vascular tone, and neuroinflammatory regulation. Mechanistic studies establish that miR-132 restricts TAU hyperphosphorylation by directly silencing TAU-directed kinases (GSK3B, MAPK1, MAPK3, ITPKB); fine-tunes TAU isoform ratios by targeting the splicing regulator PTBP2; and dampens amyloid precursor protein processing via ITPKB repression, which in turn reduces BACE1 activity. Furthermore, miR-132 acts as an anti-inflammatory rheostat by targeting EP300, IRAK1, IRAK4, and ERK1, curbing deleterious neuroinflammation. Yet, despite this critical pathophysiological role, the precise molecular drivers that differentially silence miR-132 while sparing miR-212 remain almost completely uncharacterized. Re-establishing physiological miR-132 levels represents a multifaceted therapeutic opportunity to simultaneously enhance cognitive performance and halt neurodegenerative decline (Salta and De Strooper, 2017).

In addition to the defined pathways reviewed here, several overarching questions remain unresolved. First, we lack a systematic, genome-wide census quantifying the distribution of these regulatory strategies across the 159 known human miRNA clusters. It is unclear what percentage of clusters utilizes alternative promoter initiation, structural versus protein-mediated cluster assistance, or dedicated epitranscriptomic modifications. Such benchmarking is essential to establish whether these mechanisms represent broad regulatory rules or merely reflect outlier paradigms spotlighted because of their pronounced experimental phenotypes.

Second, multi-tiered crosstalk within a single polycistronic unit remains virtually uncharted. Whether alternative transcription, splicing, structural assistance, RBPs, and epitranscriptomic writers/erasers act orthogonally, additively, or competitively is unknown. The intra-transcript combinatorial code of multiple epitranscriptomic marks is similarly unaddressed.

Comparative analyses across taxonomic groups also remain fragmentary. In plants, miRNA clusters are markedly rarer and are composed predominantly of homologous members of the same miRNA family (Li and Mao, 2006; Sunkar and Jagadeeswaran, 2008), suggesting that their principal function is to increase miRNA dosage rather than diversify regulatory outputs. This observation raises the possibility that differential regulation, or decoupling, of individual miRNAs within clusters is more characteristic of genomes containing functionally heterogeneous clusters. Accordingly, the evolutionary forces that shape miRNA clusters and the molecular mechanisms that enable their differential regulation may differ substantially among taxonomic groups. Testing this hypothesis will require comparative analyses of the regulatory elements governing homologous and non-homologous miRNA clusters across diverse taxa.

A nascent yet conceptually compelling frontier is the competitive trade-off between nuclear miRNA biogenesis and the cytoplasmic translation of primary-transcript-encoded micropeptides (miPEPs). Pri-miRNAs were long regarded as exclusively non-coding transcripts. However, recent studies have shown that the 5′regions of many plant pri-miRNAs contain short open reading frames that encode regulatory micropeptides, termed miPEPs (Lauressergues et al., 2022). This finding parallels the emerging evidence that some circRNAs, which also long considered exclusively non-coding regulatory transcripts, can undergo translation (Khan et al., 2022). Limited evidence also supports the existence of miPEPs in human cells; for example, miPEP133 is a microprotein encoded by pri-miR-34a (Kang et al., 2020). In plants, miPEPs derived from the lead miORF (such as those in pri-miR-171b and pri-miR-165a) engage in auto-regulatory positive feedback, augmenting host pri-miRNA transcription (Lauressergues et al., 2015; 2022). In human systems, however, this regulatory logic is not broadly conserved, as ectopic expression of human miPEP155, miPEP497, or miPEP200a fails to modulate host miRNA abundance (Prel et al., 2021). Although the coding potential of individual pri-miRNAs has been established in plants and has also been reported in human cells, the relationship between miRNA biogenesis and miPEP translation (particularly within polycistronic clusters) remains largely unexplored. Nuclear processing of a pri-miRNA by the Microprocessor involves endonucleolytic cleavage of the transcript and would therefore be expected to preclude its subsequent translation. Conversely, export of an intact pri-miRNA transcript to the cytoplasm could permit miPEP translation. This framework suggests that miPEP translation and pri-miRNA processing may compete for the same polycistronic transcript. However, available evidence from plants supports a possible alternative scenario. Predominantly truncated transcripts generated through alternative transcription initiation or termination, as well as alternatively spliced isoforms lacking pre-miRNA hairpins, appear to be translated. In contrast, longer transcripts are retained in the nucleus and serve as substrates for mature miRNA biogenesis (Lauressergues et al., 2022). Thus, the choice between miPEP translation and miRNA production may not necessarily reflect direct competition for an individual transcript molecule; instead, it may be established through alternative transcriptional and post-transcriptional processing pathways. Whether similar mechanisms operate in animals or humans remains unknown. The molecular determinants governing the choice between pri-miRNA processing and miPEP translation, as well as the nature of any regulated molecular switch, also remain to be elucidated. RBPs and epitranscriptomic modifications discussed in this review as regulators of miRNA processing may conceivably also contribute to these RNA-fate decisions, but this possibility requires direct experimental validation.

Looking ahead, the foremost challenge in this field is to transition from cataloging descriptive mechanistic vignettes (often derived from individual clusters and specific cell lines) toward predictive biology. Developing unified quantitative models that integrate primary sequence architecture, structural topology, RBP interactomes, and epitranscriptomic landscapes will allow us to accurately forecast the direction and extent of differential miRNA biogenesis across distinct cellular contexts. Collectively, establishing such predictive mastery over polycistronic regulation will unlock entirely new avenues for precision therapeutic intervention in human disease.
